# Small molecules as arthropod kinin receptor antagonists, feeding modulators, or novel mosquitocidal agents

**DOI:** 10.1002/ps.70764

**Published:** 2026-04-10

**Authors:** Bianca M. Henriques‐Santos, Patricia V. Pietrantonio

**Affiliations:** ^1^ Department of Entomology Texas A&M University College Station TX USA

**Keywords:** attractive toxic sugar bait (ATSB), feeding stimulants, GPCR, mosquitocidal molecules, mosquito kinin receptor, small molecule pharmacology

## Abstract

**BACKGROUND:**

*Aedes aegypti* and *Culex quinquefasciatus* mosquitoes are primary vectors for numerous human and animal pathogens and each pose significant global health threats. The worldwide problem of insecticide resistance prompted the search for novel targets and ‘lead’ insecticidal chemistries for these two species.

**RESULTS:**

The insecticidal activity and other biological effects of selected small molecules derived from an original random in‐house chemical library of 20 000 compounds were investigated against mosquitoes. A multipronged approach included a cell‐based recombinant *Aedes* kinin receptor screen for potential antagonists, and the structure–activity relationships. Out of the 88 small molecules investigated, seven kinin receptor full antagonists were identified, among which the small molecule SACC‐0048555 was myoinhibitory of the mosquito hindgut contraction, increased female sugar‐feeding behavior, and potentiated mortality by malathion, all consistent with antagonism of the kinin system. Two identified structurally related molecules, SACC‐0039590 and SACC‐0428788, were not kinin receptor antagonists but were adulticidal for both mosquito species at sub‐micromolar levels when applied topically and through tarsal contact, and disrupted feeding when applied at a concentration that kills 25% of the tested population (LC_25_).

**CONCLUSION:**

The activity of these small molecules, and their likely novel mode of action, offer a promising pathway for developing feeding stimulants for attractive targeted sugar baits and are chemical leads for future insecticide development. © 2026 The Author(s). *Pest Management Science* published by John Wiley & Sons Ltd on behalf of Society of Chemical Industry.

## Graphical abstract

A two‐pronged screen of small molecules – (1) on recombinant kinin receptor and (2) through topical application – identifies kinin receptor antagonists, feeding behavior modulators, and new mosquitocidal molecules.

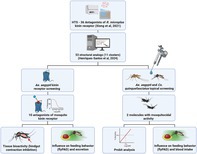

**ABBREVIATIONS**
GPCR: G protein‐coupled receptor
HTS: high‐throughput screen
IC_50_
: half‐maximal inhibitory concentration
IGKN‐G12: recombinant *Aedes aegypti* kinin receptor cell line
KR: kinin receptor
LC_25_
: concentration that kills 25% of the tested population
LC_50_
: median lethal concentration
LD_50_
: median lethal dose
RME: rapeseed oil methyl ester
V/O: vector‐only cell line

## 1 INTRODUCTION

*Aedes aegypti* is the primary vector for several human illnesses, including dengue fever, Zika, yellow fever, and chikungunya.^1^ Despite extensive efforts to control *Ae. aegypti* populations, their development of insecticide resistance, and the limitations of current control strategies present significant challenges to effective vector management.^2^ Novel, small, synthetic molecules targeting specific molecular pathways in mosquitoes have garnered attention for their potential efficacy and reduced environmental impact.^3^ The recognition of G protein‐coupled receptors (GPCRs) as key mediators of insect physiology has sparked interest in exploiting these receptors as novel targets for insecticide development.^4^, ^5^, ^6^ GPCRs are membrane receptors that play crucial roles in transducing extracellular signals and regulating various physiological processes in insects. Among the insecticide classes, only the formamidines represented by the acaricide/insecticide amitraz target a GPCR. The IRAC insecticide classification places amitraz in group 19, with the octopamine receptor as its target.^7^ Amitraz activates the tick octopamine and tyramine GPCRs.^7^, ^8^

Arthropod kinin receptors (KRs)^9^, ^10^ have emerged as promising GPCR targets. This is due to their pivotal role in regulating key physiological processes in insects, including feeding behavior^11^ and digestive enzyme secretion,^12^ sugar taste perception,^13^ diuresis,^14^ hindgut contractions,^15^ and reproduction.^16^ In ticks, kinins neuropeptides stimulate midgut peristalsis^17^ (for reviews see Nässel^16^ and Nässel and Wu^18^). *Aedes* kinins activate intracellular signaling through the G_q_ pathway, releasing intracellular calcium.^10^ However, since the natural peptides cannot be directly used for pest control due to insufficient cuticular penetration, susceptibility to degradation by endogenous peptidases as well as cost,^19^, ^20^, ^21^, ^22^ small molecules can be exploited as surrogate ligands for these ‘druggable’ receptors. Furthermore, the size of the minimal active core of the kinin peptide is small, ranging from 5–6 amino‐acid residues depending on the arthropod species,^23^ which increases the likelihood of finding small molecules as agonists or antagonists.

The search for novel insecticidal compounds can entail thoroughly investigating diverse chemical libraries through high‐throughput screening (HTS) methods, whether conducted in controlled laboratory conditions using target‐based approaches (*in vitro*) or within living organisms (*ex* and *in vivo*).^24^ Several small molecule target‐based screening efforts have focused on GPCRs in mosquitoes and ticks, such as for the dopamine,^25^, ^26^, ^27^, ^28^ neuropeptide Y‐like^29^ and kinin^30^ receptors. For mosquitoes, the Kir1 channel^31^ and the enzyme acetylcholinesterase^32^ have also been targeted. Alternatively, the *in vivo* method has primarily concentrated on pinpointing neurotoxic compounds causing mortality in both insect larvae^33^, ^34^ and adults.^35^, ^36^, ^37^, ^38^

In a previous report^30^ we screened against the tick recombinant KR 20 000 synthetic small molecules from a random, high chemical diversity in‐house library of 150 000 molecules, which yielded 36 antagonists with different potencies. Herein we first tested against the mosquito *Ae. aegypti* recombinant KR 35 of those identified antagonists^30^ and 53 of their structural analogs,^39^ identifying molecules with diverse bioactivity. This study presents a comprehensive pipeline for identifying small molecules with antagonistic activity on the *Ae. aegypti* KR or those that are mosquitocidal. Our goals were four‐fold: to (i) identify chemistries that disrupt KR signaling, (ii) assess the effect of the identified bioactive GPCR modulators on mosquito feeding behavior and physiology, (iii) bring innovation to insecticides targeting vectors by assessing the contact toxicity of other novel small molecules, including their sublethal effects on blood‐feeding behavior, and finally, (iv) explore the potential of these chemistries for mosquito control. For this, we utilized validated bioassays^38^, ^40^, ^41^ and reagents^13^, ^30^, ^42^ directly linked to the known function of kinins in mosquitoes in diuresis, hindgut contraction, and sugar feeding to verify the effect of these molecules on the mosquito kinin signaling system and on mosquito survivorship *in vitro* and *in vivo*.

## 2 MATERIALS AND METHODS

### 2.1 Chemicals

The complete list of the 88 small molecules tested is shown in Supporting Information S4 Table S1. This molecule list includes the SACC‐XXXXXXX identifier automatically generated by the CDD (Collaborative Drug Discovery) Vault (www.collaborativedrug.com, Burlingame, CA, USA), Chemical Abstract Services (CAS) numbers, SMILES (Simplified Molecular Input Line Entry System), vendor, and vendor reference number. Structural clusters are shown in  Supporting Information S4 Table S2. The original 36 antagonists were a part of the Texas AgriLife Research small molecule library in the laboratory of James Sacchettini, designated SAC‐2 library. Analogs were identified through searches in the SciFinder database (CAS SciFinder) accessed through Texas A&M University (TAMU) using the original 36 tick KR antagonists as reference Structures.^30^ Molecules were considered analogs if they exhibited at least 80% structural similarity to the original antagonists, resulting in the identification of 53 additional compounds grouped into 11 structural families.^39^ Molecules in the library and structural analogs have apparent stable structures, making them less prone to hydrolysis, and are classified as drug‐like molecules according to molecular weight, lipophilicity, number of hydrogen bond donors, and number of hydrogen bond acceptors (Lipinski's Rule of Five^43^).

### 2.2 Insect rearing

Non‐blood‐fed female mosquitoes of the insecticide‐susceptible Liverpool strain of *Ae. aegypti* (L.) or the Sebring strain of *Culex quinquefasciatus* Say, were used for all bioassays. Females were 2–5 day‐old for topical application of small molecules and 7–14‐day‐old for feeding behavior bioassays. Mosquitoes were maintained in an incubator at 28 °C and approximately 80% humidity, with a 16 h:8 h light/dark photo cycle. Ground fish food (Tetra, Blacksburg, VA, USA) was provided throughout the aquatic developmental stages, and 10% sucrose solution was supplied *ad libitum* during the adult stage.

### 2.3 Cell maintenance

The *Ae. aegypti* KR is stably expressed in the recombinant CHO‐K1 clonal cell line designated IGKN‐G12.^30^ CHO‐K1 cells transfected with the empty plasmid (pcDNA™3.1; Invitrogen, Waltham, MA, USA) were used as control and are referred to as ‘vector‐only’ (V/O). IGKN‐G12 or V/O cells were cultured in T‐75 flasks with a selective medium (F‐12 K medium containing 10% fetal bovine serum (FBS) and 800 μg/mL of G418 sulfate) for one to two passages before the ‘dual‐addition’ calcium fluorescence assay.^44^ All cells were kept at 37 °C and 5% carbon dioxide (CO_2_) in a humidified incubator.

### 2.4 Drug plate preparation for dual addition assay

Dose–response analysis of each small molecule (*n* = 88, as in 35^30^ plus 53 structural analogs^39^) was performed using ten concentrations, from 25 μm to 12.7 pm, as final concentrations in the assay plate (Section 2.5). However, first a drug plate had to be prepared. For that, each well of the 96‐well drug plate was filled with 56 μL of Dulbecco's phosphate‐buffered saline (DPBS, 650201; Greiner, Kremsmünster, Austria) with 10% (*v/v*) dimethyl sulfoxide (DMSO). Then, 22 μL of DPBS with 1.82% DMSO was added only into the wells of the second column in the 96‐well plate. Lastly, 2 μL of each molecule stock solution at 10 mm in 100% DMSO (D‐2650; Sigma‐Aldrich, St Louis, MO, USA) were also added to the second column wells, which constituted the starting concentration (250 μm) for serial dilutions in this stock drug plate. As every well in the drug plate already contained DPBS with 10% DMSO, the serial dilution was achieved starting by transferring 14 μL from column 2 wells into 56 μL DPBS 10% (*v/v*) DMSO into column 3 and then continuing the transferring of 14 μL successively from each column through column 11, resulting in a serial dilution factor of 1:5. The dilutions were carried out using a Viaflo liquid handling system (96/300 μL; Integra Biosciences, Hudson, NH, USA). This drug plate was used as a stock from which to transfer 11 μL of each molecule concentration into the assay plate, as explained in Section 2.5. Therefore, the final DMSO concentration in the assay plate was 1%, for just 5 min, a concentration previously determined not to cause cytotoxic effects on CHO‐K1 cells, with comparable viability to the negative control.^30^, ^44^, ^45^, ^46^, ^47^, ^48^ This is significantly below the cytotoxic threshold for CHO‐K1 cells, which exhibit a half‐maximal inhibitory concentration (IC_50_) of approximately 2.7% DMSO, only after a much longer 100‐h incubation.^45^

To confirm their antagonistic activity and estimate IC_50_ values, new drug plates were prepared for the ten selected antagonists using a larger dilution factor (1:2) to focus on the active dilution range resulting in ten concentrations from 25 μm to 48.8 nm. For both drug plate designs, columns 1 and 12 contained DPBS with 10% DMSO only as negative controls.

### 2.5 Kinin receptor ‘dual addition’ calcium fluorescence assay

The intracellular calcium fluorescence endpoint assay was adapted from previous protocols^30^, ^44^ by adjusting volumes for the 96‐well plates instead of 384‐wells (final volume 100 μL *versus* previous 25 μL). Briefly, when cells reached 90% confluency,^49^ they were trypsinized and suspended in F‐12K medium containing 1% FBS and 400 μg/mL G418 sulfate at a density of 4 × 10^5^ cells/mL. The cells were seeded in 96‐well black plates with clear bottom (655090; Greiner) that had been coated with poly‐d‐lysine (P6407; Sigma‐Aldrich). Using the Viaflo liquid handling system equipped with a 96‐pipetting head (96/300 μL), 100 μL of the mentioned cell suspension was dispensed into each of the wells. This liquid handling system was used for all pipetting steps. Plates were incubated overnight at 37 °C and 5% CO_2_. After cell incubation, the loading dye (1×) was prepared according to the manufacturer's instructions by diluting FLUOFORTE (ENZ‐51017; Enzo Life Sciences, East Farmingdale, NY, USA) dye into the assay buffer (1:1000), consisting of 1× HHBS (Hank's buffer with 20 mm HEPES) and dye efflux inhibitor (9:1). The medium was removed from the plate by inverting it onto a paper towel and replaced by 100 μL of loading dye (1×). The plate was placed in the incubator for 30 min, and left to equilibrate in the dark at room temperature (RT) for another 30 min.

The ‘dual addition’ assay was conducted as described previously.^30^, ^44^ Initially, the plate containing cells and 100 μL of assay buffer per well was read to obtain the control background signal (in relative fluorescence units, RFU) in endpoint reading mode. Subsequently, for the first addition, 11 μL of the test compounds (from 25 μm to 12.7 pm) were transferred from the drug plate into the assay plate containing the cells, and the fluorescence was assessed. For the second addition, after 5 min incubation period, 12 μL of kinin agonist 1728 ([Aib]FF[Aib]WGamide, custom synthetized by Royobiotech Co., Ltd, Shanghai, China)^13^, ^42^, ^50^ were dispensed into each well to a final concentration of 1 μm, and the fluorescence was again measured. The fluorescent intensity was measured at excitation/emission wavelengths of 495/525 nm in plate endpoint mode using a Clariostar plate reader (BMG Labtech, Ortenberg, Germany). The plate was read in the flying mode from both forward and reverse orientations to compensate for the decrease in signal during the reading time. The responses were represented as the average of the two values obtained, normalized by subtracting the background signal reading. The small molecule inhibition of the fluorescence agonist response was calculated as the ratio between the response generated by cells treated with the compounds and the control response. The control response consisted of the RFU read after the kinin agonist 1728 was added in the second addition of the assay to cells that had received ‘solvent only’ in the first addition. To ensure that the induced fluorescence was not due to unspecific activation of endogenous CHO‐K1 cell receptors, the same compounds were tested on V/O cells.

### 2.6 Topical and tarsal contact bioassays, and heat stress after spraying

For single‐dose screening bioassays of the 88 small molecules, molecules were reconstituted in DMSO (100 mm stock). Solubility at a high concentration, such as 100 mm, was verified considering the partition coefficient, which measures how much of a solute dissolved in the water portion *versus* an organic portion. Stock molecules in DMSO were diluted in acetone (≥ 99.5% pure,179124; Sigma‐Aldrich) to a working solution of 1.25 mm. For this, acetone was previously ‘dried’ overnight by pouring it over 4 Å molecular sieves (20859‐0; Sigma‐Aldrich), and the sealed container was kept in a desiccator until usage.^51^ For thorax topical applications, three groups of 20 female mosquitoes were treated either with the test molecules, solvent‐only (negative control), or permethrin (45614; Sigma‐Aldrich) at 100 μm (positive control). For this, females were anesthetized for 30 s with CO_2_ in a WHO tube and transferred to a cold tray that had been kept at −20 °C (4650; Sakura Finetek, Torrance, CA, USA), where they were distributed with the dorsal thorax exposed. A 0.2 μL droplet of the working solution^38^ was placed onto the pronotum using a 10 μL syringe (7653‐01; Hamilton, Reno, NV, USA) coupled with a 26s‐gauge needle (7804‐04; Hamilton) using a manual repeating dispenser (83700; Hamilton). Treated mosquitoes were transferred immediately to 120 mL glass jars containing a 1.5 mL microtube with a 10% (*w/v*) sucrose solution closed with a cotton ball to allow mosquitoes to feed *ad libitum*. The jars were covered with voile fabric held with elastic bands and maintained in an incubator as described in Section 2.2. Mosquito mortality was scored 24 h after treatment according to the World Health Organization (WHO)^52^ definition, that is, a mosquito is classified as dead or knocked down if it is immobile or unable to stand or take off.

Upon confirmation of molecule antagonism in the recombinant receptor assay (see Section 3.1), bioassays were repeated for each of the ten selected antagonists at 1 mm with the addition of rapeseed oil methyl ester (RME, MERO® 81.4% *w/w*; Bayer, Reading, UK). A 0.392 mg/mL RME (~400 ppm) in acetone solution was used to prepare the working solutions, which were applied to the mosquito thorax for screening as described earlier. The RME concentration was that of a typical field application rate and stands well below the maximal allowable rate (1% ≈ 9 mg/mL).^53^ Additionally, RME was also used to perform serial dilutions of the molecules with adulticidal activity, namely SACC‐0039590 and SACC‐0428788, and solutions were similarly applied on the female's thorax for the concentration–mortality curves, as described earlier. Solution concentrations and volume applied (0.2 μL) on mosquitoes were used to calculate doses per female and to estimate the median lethal dose (LD_50_) in nmoles per female.

To assess the tarsal contact activity in vial bioassays serial dilutions were prepared using acetone containing RME at 100 ppm for *Ae. aegypti* and 50 ppm for *Cx. quinquefasciatus* as solvent. Solutions were dispensed into 20 mL glass scintillation vials (500 μL) which were placed on a hot dog roller inside a chemical hood for 15 min. The rotation was used to evenly coat the inside of the vial and dry the solvent without heat. The inside of the caps was treated with 50 μL of the respective solutions and allowed to dry.^41^ Females of *Ae. aegypti* or *Cx. quinquefasciatus* (2–5 days old) were anesthetized on ice for 4 min and ten mosquitoes were placed in each vial. Vials were closed then kept in an incubator at 28 °C and approximately 80% relative humidity for 24 h, when mortality was assessed. Vials were opened inside a cage and mosquitoes unable to fly or stand were considered dead.

To evaluate the effect of high‐temperature stress on treated females, 3–5‐day‐old unfed *Ae. aegypti* females were sprayed with acetone containing 0.392 mg/mL RME or one of the mosquitocidal molecules, SACC‐0039590 or SACC‐0428788, (in acetone containing 0.392 mg/mL RME) at a sublethal concentration (concentration that kills 25% of the tested population, LC_25_ = 0.2 mm) using a handheld atomizer as aforementioned, and allowed to recover for 1 h. Females were then anesthetized with CO_2_ and placed in 50 mL flat bottom vials (ten females each) and allowed access to a 10% sucrose solution, as described earlier, for 1 h. Then, vials were either kept at RT or placed in an incubator at 41 °C and approximately 55% humidity. The knockdown effect was assessed every 5 min for 30 min.^54^

### 2.7 Feeding behavior bioassays, antidiuretic activity, and meal consumption

To assess the effects of ten antagonists on the feeding behavior of *Ae. aegypti* females, the adapted flyPAD automated monitoring system was used as described previously.^40^ Chambers which are divided into four arenas with two wells each for adding diets were placed onto a slide warmer (Barnstead/Lab‐Line, Dubuque, IA, USA) at 37 °C for feeding assays. Then, up to 4 μL of the meals were pipetted into each well (channel) following a non‐choice design, in which both channels within the same arena received the same meal. Meals consisted of 10% sucrose containing 0.002% (*w/v*) fluorescein sodium salt (0681; VWR, Radnor, PA, USA), as final concentration, or defibrinated sheep blood (HemoStat Laboratories, Dixon, CA, USA) containing 0.002% fluorescein and ATP (A2383; Millipore Sigma, Bedford, MA, USA) at 1 mm. For meals supplemented with small molecules, stocks (100 mm) in DMSO were applied to the meals for a final concentration of 1 mm. Control meals had only DMSO added at the equivalent concentration of 1% (*v/v*). Females were anesthetized with CO_2_ for 30 s, and one female was transferred to each arena. Feeding behaviors were recorded for 30 min. Capacitance and video were captured using the Bonsai data stream processing package available at http://www.flypad.pt.^55^, ^56^ Video monitoring was performed using a Blackfly camera (BFS‐U3‐16S2C‐CS; FLIR Integrated Imaging Solutions, Inc., Richmond, BC, Canada). Subsequently, signal processing and data analysis were done in MATLAB (Mathworks Inc., Portola Valley, CA, USA). At the end point of the assays, females were collected for ingested meal volume estimation as described previously.^40^ Feeding behaviors elicited by the different molecules were analyzed as a ratio of their respective control group output to allow comparisons of molecules’ effects.

To evaluate the potential antidiuretic effect of KR antagonist SACC‐0048555, this molecule was diluted at 1 mm in a 10% sucrose solution containing 0.1% Evans blue dye (A16774; Alfa Aesar, Ward Hill, MA, USA). Twenty females that had been starved for 24 h were anesthetized and placed into 100 mm × 15 mm Petri dishes (351029; Corning, Corning, NY, USA) containing a single 50 μL drop of either sucrose only or sucrose with the test molecule.^13^ Females were allowed to feed on the drop for 5 h and, at the endpoint, the total number of excreted drops, observed as blue deposits on the Petri plates, was counted. Females were collected to estimate the remaining meal volume in their guts after the 5 h observation period.

To investigate the potential feeding stimulant effect of 8555, feeding assays with malathion were similarly conducted but with ten females per plate. Mosquitoes were offered four meals: 10% sucrose only as negative control; 10% sucrose with malathion at 0.66%, which is the LC_50_ for susceptible *Ae. aegypti*;^57^ or 10% sucrose 8555 at 1 mm with 0.66% malathion. Mortality was assessed every hour for 5 h.

The sublethal effects of the two mosquitocidal molecules on the blood volume ingested and blood‐feeding behavior of females, were evaluated after topical applications performed as described (Section 2.6). Molecules SACC‐0039590 and SACC‐0428788 as treatments, and SACC‐0412060, as a negative control, were prepared at 0.2 mm, the LC_25_ for the most effective molecule, SACC‐0039590, using 0.392 mg/mL RME in acetone as solvent. After treatment, mosquitoes were transferred to glass jars and allowed to recover for 1 h without providing them with the sucrose solution. Females were then offered blood containing 0.002% (*w/v*) fluorescein in artificial feeders (‘glytubes’), as described in the literature.^58^ After 30 min mosquitoes were collected and the remaining volume ingested was estimated (Section 2.8).

To assess the feasibility and efficacy of the spray application, the assay was repeated using 10 mL atomizer spray bottles (ZbFwmx; Amazon, Seattle, WA, USA) to apply SACC‐0039590, SACC‐0428788, and SACC‐0412060 at 0.2 mm in acetone containing 0.392 mg/mL RME.^38^ Females were placed in a 20.5 cm^3^ cage and were sprayed with 2 mL of these solutions. Then, after 1 h of recovery, responsive/alive mosquitoes were collected using a mechanical aspirator (13500; Clarke, St Charles, IL, USA) and placed in flyPAD arenas to evaluate their blood‐feeding behavior. Alternatively, mosquitoes were kept in cages and offered blood in an artificial feeder,^58^ then the meal volume was quantified (Section 2.8).

### 2.8 Estimation of remaining ingested meal volume

The total volume of sucrose or blood remaining in each female from experiments in Section 2.7 was estimated as previously described^59^ because the respective meals had been supplemented with 0.002% (*w/v*) fluorescein. After feeding assays, mosquitoes were collected and placed in 1.2 mL microtubes (19560 and 19566; Qiagen, Germantown, MA, USA) containing one 2.8 mm ceramic bead (19‐646; Omni International, Kennesaw, GA, USA) and 100 μL of phosphate‐buffered saline (PBS). Samples were disrupted with a TissueLyser II (Qiagen) for 30 s using plate adapter sets (69984; Qiagen). Racks containing microtubes were briefly centrifuged for 20 s at 600 × *g*. For every assay, a standard curve was prepared by adding 10 μL of the meal containing 0.002% fluorescein to 390 μL of PBS and serially diluting it at a 2× dilution rate. For each dilution, 100 μL were transferred to a 1.2 mL microtube with one 2.8 mm ceramic bead and a non‐blood fed female and homogenized as indicated earlier. For both samples and the standard curve, 20 μL of the homogenate was transferred to a 96‐well black/clear bottom plate (655090; Greiner) containing 180 μL of PBS. The fluorescent intensity was measured using a ClarioStar plate reader at wavelengths 485/520 nm excitation/emission. The meal volumes were calculated as a ratio between the RFU corresponding to each female homogenate sample and the standard curve slope (RFU/μL).

For females analyzed for urine excretion, the remaining meal volumes were quantified based on the amount of Evans blue in the sample, adapted from Sakuma and Kanuka.^60^ Collected mosquitoes were placed in microtubes with one ceramic bead as before but with 230 μL of PBS. Samples for standard curves were prepared by adding 10 μL of the 10% sucrose with 0.1% Evans blue in 450 μL of PBS, serially diluting the sample at a 2× rate, and finally adding a female to each tube. Samples from treatments and standard curves were homogenized as described earlier, and racks were centrifuged for 3 min at 2500 × *g*. Homogenate supernatants (100 μL) were transferred to a 96‐well black/clear bottom plate and the fluorescent intensity was measured at 520/580 nm. Volumes were calculated in the same manner.

### 2.9 Validation of antagonistic myotropic activity

To assess the effect of the antagonist small molecules on the hindgut contraction, 3–5‐day‐old females were used following a described protocol in the literature.^30^ Briefly, the hindgut was transferred into a drop of 30 μL Ringer's solution in a well of a 24‐well‐plate. Paraffin oil (~500 μL) was added to each well to cover the saline and prevent evaporation. Each small molecule solution (3× concentrated in Ringer's solution; 15 μL of 300 μm) or buffer only was added for a final concentration of 100 μm. After 5 min incubation, kinin agonist analog 1728 solution (4× concentrated in Ringer's solution) was added to the drop with the tissue for a final concentration of 10 μm. After 1 h of incubation, the tissue was filmed for 30 s using an INFINITY5 camera (Teledyne Lumenera, Ottawa, Canada) mounted on an SZ60 stereomicroscope (Olympus, Center Valley, PA, USA). Videos were analyzed for activity generated by the hindgut contractions within the area using video tracking software Ethovision XT 17 (Noldus, Wageningen, The Netherlands).^17^, ^39^

### 2.10 Statistical analysis

All experiments were independently repeated at least three times and the resulting biological replicate numbers (*n*) were indicated in the figure legends. All statistical analyses were performed using GraphPad Prism v9.5 software (GraphPad Software Inc., San Diego, CA, USA). The dose–response curves for the antagonistic small molecules on the recombinant *Ae. aegypti* KR were calculated selecting non‐linear regression [log(inhibitor/agonist) *versus* response‐variable slope (four parameters)], with IC_50_ in μm. For hindgut contraction inhibition assays, for the nine flyPAD variables analyzed and for estimations of the remaining meal volumes, data sets were first tested for normality, and appropriate parametric or non‐parametric statistical analyses were applied accordingly. For hindgut contraction inhibition assays, the rank among medians of the different small molecules was analyzed by the non‐parametric Kruskal–Wallis test, followed by Dunn's multiple comparisons test. The results were presented as the mean ± standard error of the mean (SEM). For flyPAD feeding behavior assays, ranks were compared using the non‐parametric Mann–Whitney test, and the results were presented for individual females along with the mean ± SEM. To assess the remaining meal volume of topically treated females, because data sets were distributed normally, results were analyzed by one‐way analysis of variance (ANOVA), followed by Tukey's multiple comparisons tests, and presented as mean ± SEM. Furthermore, *t*‐tests were used to compare mosquito mortality with different solvents. GraphPad Prism was also used to transform mosquito mortality into probits and the estimation of lethal doses/dosages (LD_25_ and LD_50_) of adulticidal molecules on mosquitoes was performed by PoloSuite (LeOra Software LLC, Parma, MO, USA).

## 3 RESULTS AND DISCUSSION

### 3.1 Identification of hit molecules on the mosquito kinin receptor

**Figure 1:**
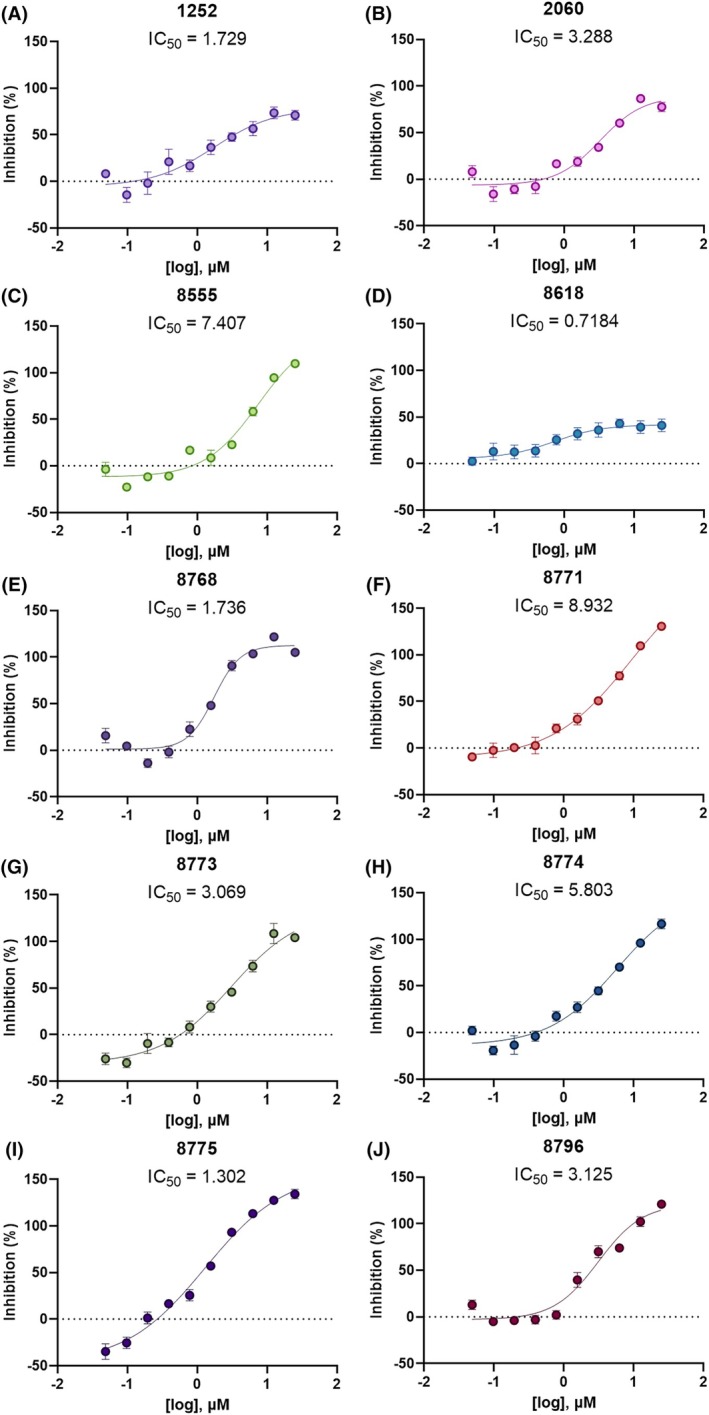
Characterization of antagonists through dose–response curves on recombinant *Aedes aegypti* kinin receptor cell line IGKN‐G12. Each compound was assessed at ten final concentrations in the assay plate, ranging from 25 μm to 48.8 nm. The inhibition percentage was calculated as a ratio of the response to 1728 in the buffer‐only wells. (A) SACC‐0121252, (B) SACC‐0412060, (C) SACC‐0048555, (D) SACC‐0018618, (E) SACC‐0428768, (F) SACC‐0428771, (G) SACC‐0428773, (H) SACC‐0428774, (I) SACC‐0428775, and (J) SACC‐0428796. The dose–response curves and half‐maximal inhibitory concentration (IC_50,_ μm) were generated by GraphPad Prism software through a non‐linear regression fit [log(inhibitor) *versus* response‐variable slope (four parameters)]. Although the 88 molecules were evaluated for agonism or antagonism through this dose–response dual‐addition assay herein, only the curves for the selected ten antagonists are shown (see Supporting Information, S4 Table S3 for additional results). Symbols are means with ± standard error of the mean (SEM). Each panel represents the mean of four independent plate assays.

Discovering novel insecticidal leads may involve screening diverse chemical libraries via HTS. Herein, 88 small molecules either previously identified as antagonists of the tick *Rhipicephalus microplus* KR through HTS^30^ or as their structural analogs after structure–activity relationship (SAR) analyses^39^ were evaluated for effects on female mosquitoes. To find molecules specific to the mosquito (*Ae. aegypti*) KR we first assessed 35 molecules antagonists of the cattle fever tick (*R. microplus*) KR^30^ and 53 of their structural analogs^39^ on the *Ae. aegypti* kinin recombinant receptor (IGKN‐G12 cell line) using a ‘dual‐addition’ calcium assay.^44^ Interest in small molecule agonists or antagonists of the KR increased after our discovery of the aversive effect on sugar feeding caused by the kinin neuropeptide analog 1728, of sequence [Aib]FF[Aib]WGamide, which acts directly shutting down the sugar neurons associated with long labellar hairs in the *Ae. aegypti* female.^13^ Each of the 88 molecules was evaluated through dose–response analyses (Supporting Information S4 Table S3). Antagonists (Fig. 1) were selected based on their ability to inhibit the cellular response to the kinin agonist 1728, showing dose‐dependent calcium release inhibition (Supporting Information S4 Table S3, red highlights). The ten best performing antagonists were selected based on their low IC_50_, specificity of the response in IGKN‐G12 *versus* V/O cells, and/or a significant IC_50_ difference between the two cell lines, namely SACC‐0121252, SACC‐0412060, SACC‐0048555, SACC‐0018618, SACC‐0428768, SACC‐0428771, SACC‐0428773, SACC‐0428774, SACC‐0428775, and SACC‐0428796, hereafter referred to by the last four digits of their identifier. Antagonistic activity was then confirmed using a narrower dilution range to target the active portion of the dose–response (Fig. 1(A)–(J), respectively). Apart from molecules 1252 (71 ± 5%, Fig. 1(A)), 2060 (77 ± 5%, Fig. 1(B)), and 8618 (41 ± 6%, Fig. 1(D)), which were partial antagonists, all other molecules inhibited 100% of the IGKN‐G12 calcium response at 25 μm. Among the full antagonists, molecule 8775 was the most potent with the lowest IC_50_ of 1.302 μm. Considering the remaining 78 molecules, nine molecules inhibited approximately 25% of the fluorescence response, 8–50%, and 2–100% but with IC_50_ > 25 μm. The other 59 molecules were not inhibitory. Despite the substantial conservation among tick and mosquito kinin sequences,^47^ only approximately 18% of tick KR small molecule antagonists also antagonized the mosquito KR, underscoring species‐specific pharmacology. Out of the 36 validated antagonists of the cattle fever tick KR, only four antagonized the mosquito KR, namely 1252, 2060, 8555, 8618 (Fig. 1(A)–(D)), among which only 8555 is a full antagonist. The analysis of the 53 structural analogs of the tick KR antagonists, out of which 20 showed antagonistic activity on the tick receptor,^39^ the IGKN‐G12 line revealed only six were antagonists of the mosquito receptor: 8768, 8771, 8773, 8774, 8775, and 8796 (Fig. 1(E)–(J)). We were unable to purchase molecule 3274 for further testing beyond the original detection in HTS.^30^

**Figure 2:**
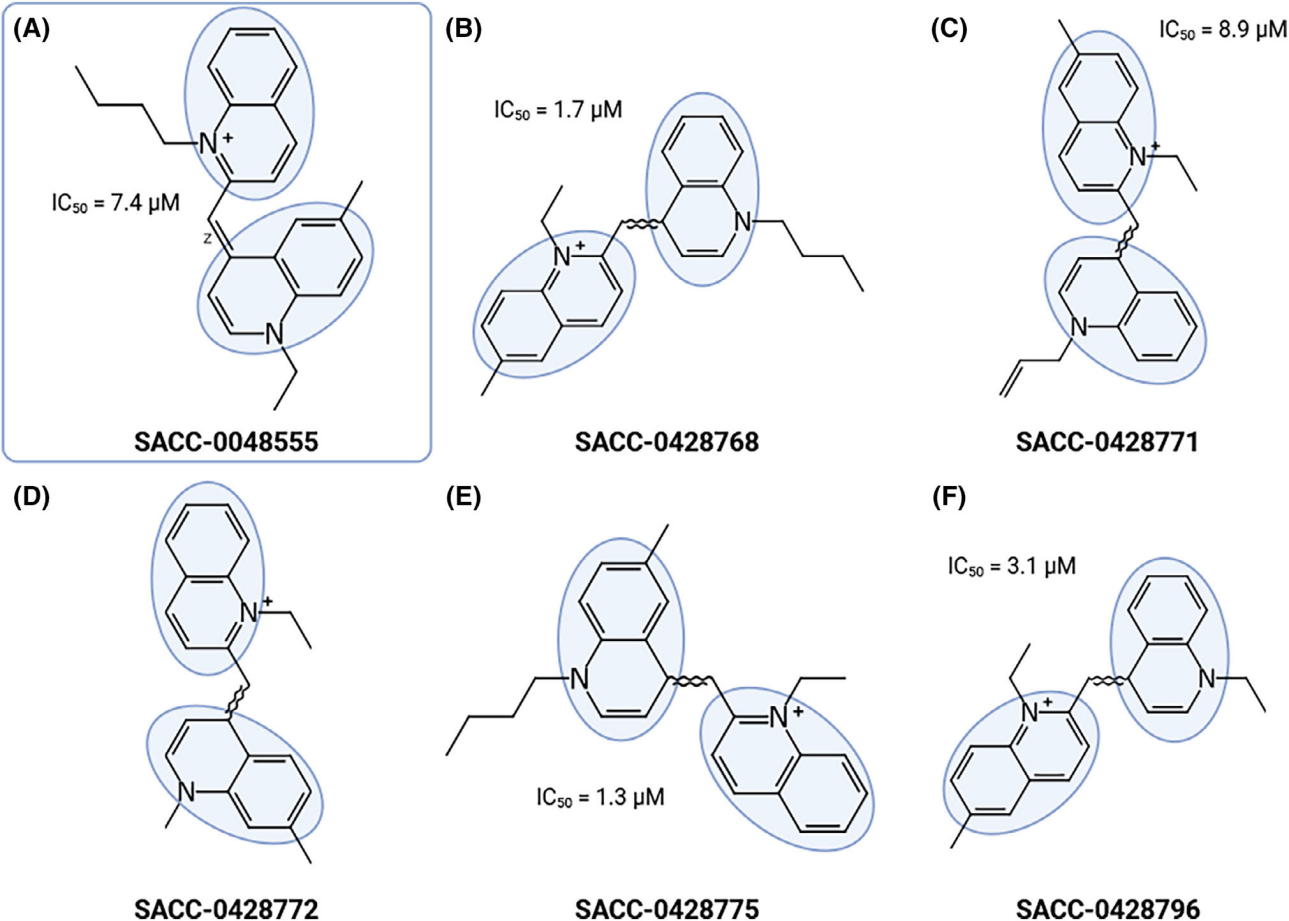
SAR analysis. Structural similarity within a cluster of *Aedes aegypti* kinin receptor antagonists and respective half‐maximal inhibitory concentration (IC_50,_ μm). ‘Parent’ molecule, SACC‐0048555 (A),^30^ and its structural analogs (B–F).^39^ Quinoline groups are highlighted in blue. (D) The molecule SACC‐0428772 did not antagonize the receptor. Created with BioRender.com.

Out of these six, four molecules belong to the same structural cluster (Fig. 2) derived from the ‘parent’ molecule 8555 (Fig. 2(A)), and structural similarities within this cluster ranged from 92% to 97%, according to SciFinder (Supporting Information  S4 Table S2).^39^ However, these six molecules (8768, 8771, 8773, 8774, 8775, and 8796) were cytotoxic to human dermal fibroblasts (HDF).^39^ Within the structural family of molecule 8555 (Fig. 2), molecules 8768, 8771, 8775, and 8796 (Fig. 2(B),(C),(E),(F), respectively) caused full KR inhibition with IC_50_ ranging from 1 to 8 μm. Molecule 8555 is a 1‐butyl‐2‐[(1‐ethyl‐6‐methyl‐4(1*H*)‐quinolinylidene)methyl]quinolinium. Quinoline is a heterocyclic aromatic compound with two rings in which carbon 1 is substituted by nitrogen. Quinoline derivatives are functional in diverse applications, such as pesticides,^61^ anti‐malarial drugs,^62^ antibacterials, and antifungals.^63^, ^64^ Even though 8555 was not cytotoxic as it did not inhibit HDF growth,^30^ the aromatic amines, known to be carcinogenic and mutagenic,^65^ are likely the reason for the cytotoxicity of its structural analogs.^39^

### 3.2 Antagonistic activity validation of small molecules on the mosquito hindgut contraction inhibition assay

**Figure 3:**
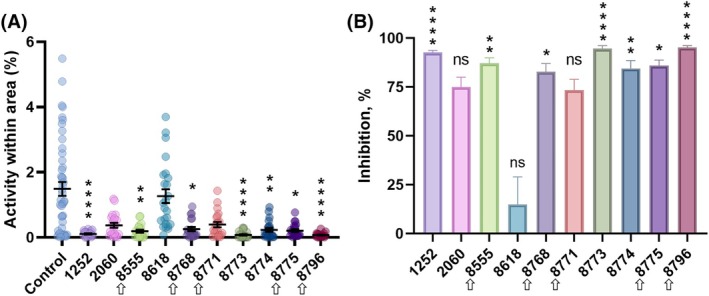
Validation of selected antagonists of the kinin receptor in the *ex vivo* hindgut contraction‐inhibition assay. Isolated hindguts from non‐blood fed females of *Aedes aegypti* were preincubated with antagonists (100 μm) or saline control for 5 min. Subsequently, the kinin agonist analog 1728 ([Aib]FF[Aib]WGamide) (10 μm) was added. Tissues were filmed for 30 s at 1 h post‐treatment. The control consisted of agonist analog only, and molecules are represented by the last four digits of their identifier (SACC‐0121252, SACC‐0412060, SACC‐0048555, SACC‐0018618, SACC‐0428768, SACC‐0428771, SACC‐0428773, SACC‐0428774, SACC‐0428775, and SACC‐0428796, respectively). Arrows denote molecules of the same structural family (SACC‐0048555). (A) Symbols represent tissue activity percentage of individual hindguts and lines are means (*n* = 47 for control and 24 for each antagonist) ± standard error of the mean (SEM). (B) Bars are mean ± SEM percentage of inhibition induced by antagonist calculated as a ratio of the average activity of the control group. Kruskal–Wallis followed by Dunn's multiple comparisons test, asterisks denote statistical significance, where one asterisk (*) indicates *P* < 0.05, two asterisks, (**) *P* < 0.01, and four asterisks (****), *P* < 0.0001.

Given that kinin peptides are myotropic on the hindgut of different insect species,^66^, ^67^ including *Ae. aegypti*.^15^ The ten antagonists that were selected based on the IGKN‐G12 cell line results were evaluated for their ability to inhibit the myotropic activity induced by the kinin agonist 1728 on the female hindgut (Fig. 3). As the EthoVision analysis software compares pixel value changes between frames, significant differences in the mean hindgut activity among treatments were discernible within the video‐captured area. More pronounced tissue contractions led to more pronounced pixel alterations in subsequent images, visually represented by automated magenta highlights generated by the software (Supporting Information S2 and S3, Video S1 and Video S2). Tissues stimulated with agonist 1728 alone had high tissue contraction activity, as seen in the extensive magenta area (Supporting Information S2, Video S1). Conversely, in the presence of the antagonist, the contraction activity was noticeably reduced (lesser magenta highlights in Supporting Information S3, Video S2). Hindguts incubated with 1728 had a significantly higher activity percentage than those preincubated with 1252, 8555, 8768, 8773, 8774, 8775, and 8796 (Fig. 3(A)) before the addition of 1728. Therefore, these seven molecules showed myoinhibitory activity on contractions of the mosquito hindgut (Fig. 3(A)), verifying their biological activity. The mean percentage of activity was used to calculate the inhibition percentage for each molecule compared to the 1728 control activity (Fig. 3(B)). As expected, molecule 8618, which inhibited only 40% of the calcium fluorescence, resulted in the least hindgut inhibition (Fig. 3(A),(B)). The tested small molecules exhibit distinct chromatic properties, and incubation with these antagonists resulted in staining of the otherwise transparent or whitish hindgut tissue, as observed in Supporting Information S2, Video S1. Molecule 8555 exhibits a deep purple coloration, and incubation of the tissue with this compound resulted in a pink staining of the hindgut, as shown in Supporting Information S3, Video S2.

### 3.3 Assessment of kinin receptor antagonists on feeding behavior

**Figure 4:**
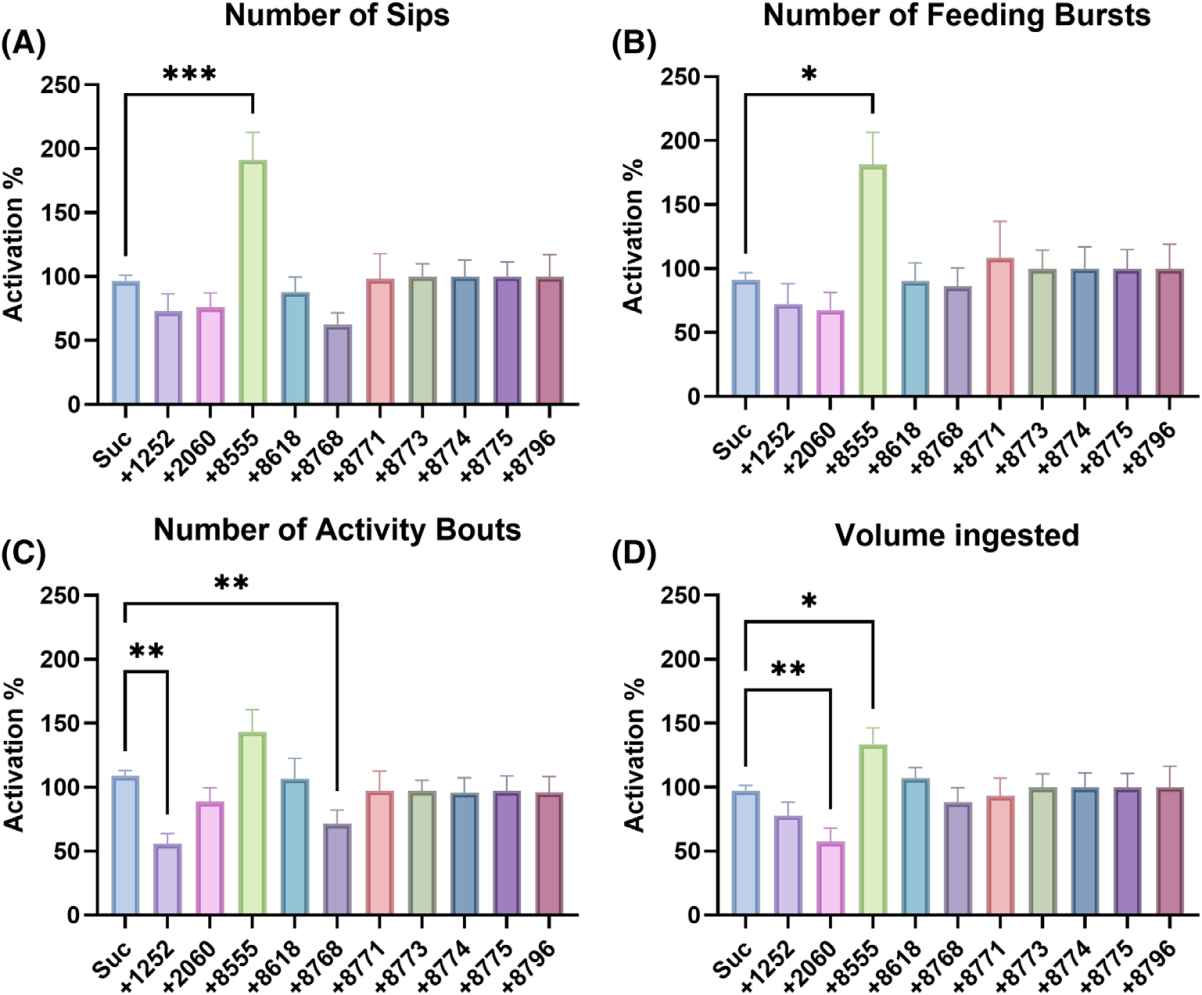
Influence of antagonists in sucrose feeding behavior and meal intake of 7–14‐days‐old female *Aedes aegypti* evaluated by flyPAD. Females were offered either 10% sucrose with 1% (*v/v*) DMSO or one of the molecules to a final concentration of 1 mm and 1% (*v/v*) DMSO, in a non‐choice manner (fluorescein included for meal quantitation). Molecules are represented by the last four digits of their identifier numbers, corresponding to SACC‐0121252, SACC‐0412060, SACC‐0048555, SACC‐0018618, SACC‐0428768, SACC‐0428771, SACC‐0428773, SACC‐0428774, SACC‐0428775, and SACC‐0428796. Data was normalized as a ratio of the treatment group to the respective control group for each specific molecule. (A) Number of sips. (B) Number of feeding bursts. (C) Number of activity bouts. (D) Volume of 10% sucrose ingested by females. Bars are mean ± standard error of the mean (SEM) percentage of activation/inhibition induced by antagonist (*n* = at least 48 females). Kruskal–Wallis followed by Dunn's multiple comparisons test, asterisks denote statistical significance, where one asterisk (*) indicates *P* < 0.05, two asterisks (**) indicate *P* < 0.01, and three asterisks (***) indicate *P* < 0.001.

**Figure 5:**
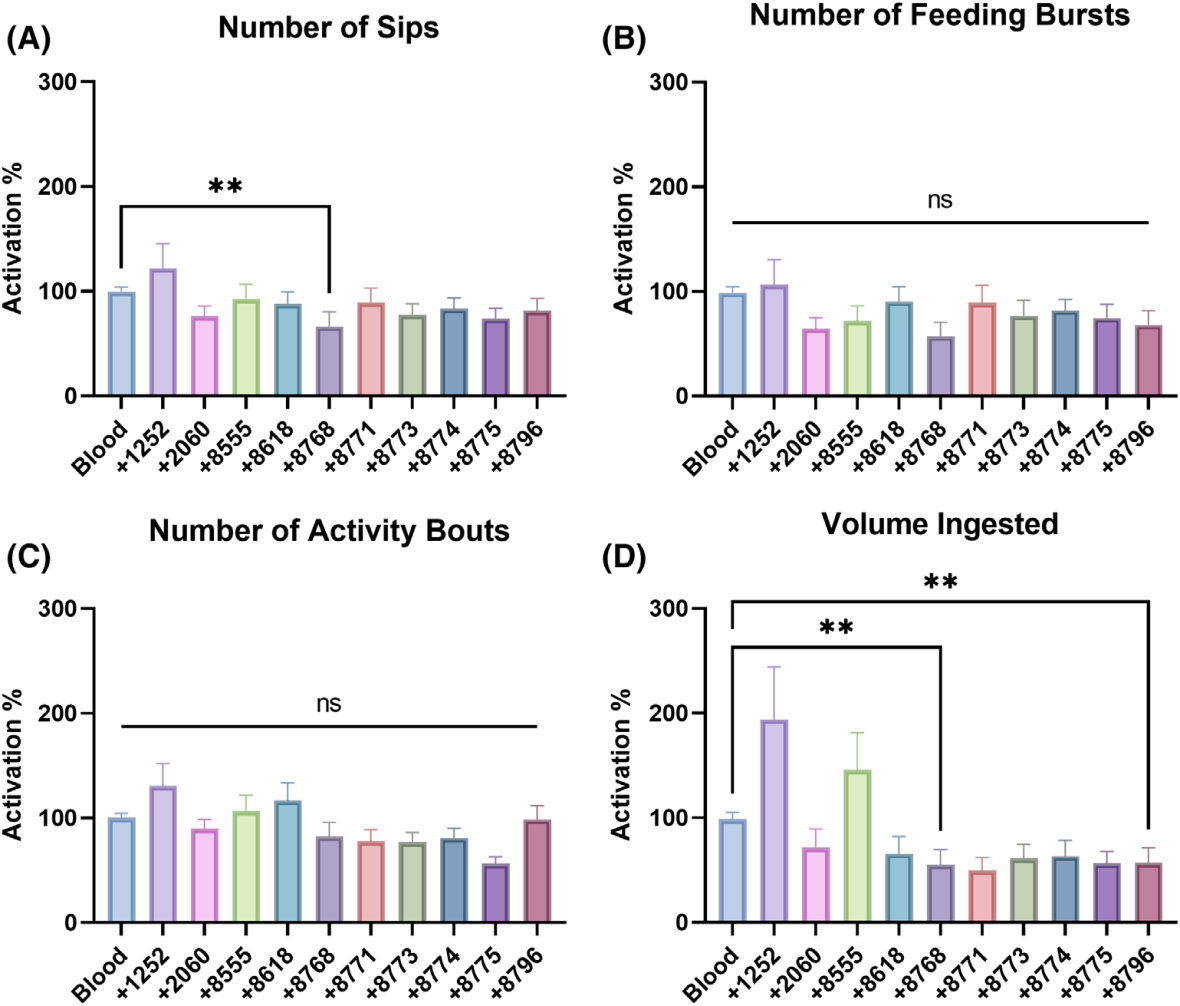
Influence of antagonists in blood feeding behavior and meal intake of 7–14‐days‐old female *Aedes aegypti* evaluated by flyPAD. Meals were prepared with sheep blood supplemented with 0.002% (*w/v*) fluorescein, for meal quantitation, and 1 mm ATP, in a non‐choice manner. Females were offered either the supplemented blood alone or with each of the molecules at the final concentration of 1 mm and 1% (*v/v*) DMSO. Molecules are represented by the last four digits of their identifier numbers, corresponding to SACC‐0121252, SACC‐0412060, SACC‐0048555, SACC‐0018618, SACC‐0428768, SACC‐0428771, SACC‐0428773, SACC‐0428774, SACC‐0428775, and SACC‐0428796. Data was normalized as a ratio of treatment group to the respective control group for each specific molecule. (A) Number of sips. (B) Number of feeding bursts. (C) Number of activity bouts accounted. (D) Volume of 10% sucrose ingested by females. Bars are mean ± standard error of the mean (SEM) percentage of activation/inhibition induced by the antagonist. Kruskal–Wallis followed by Dunn's multiple comparisons test, asterisks denote statistical significance, where two asterisks (**) indicate *P* < 0.01, and not significant (ns) indicates *P* > 0.05.

To investigate the influence of KR antagonists on feeding behavior and meal ingestion, the ten selected antagonists were added individually to either sucrose or blood meals in non‐choice assays performed using the flyPAD^40^ (Figs 4 and 5, and Supporting Information, Fig. S1). Molecule 8555 in sucrose significantly increased the number of sips by approximately 91% ± 21% (Fig. 4(A)), the number of feeding bursts by approximately 81% ± 15% (Fig. 4(B)), and the volume of sucrose ingested by approximately 34% ± 13% (Fig. 4(D)). Molecule 8555 did not significantly increase the number of feeding bouts (~43% ± 17%) (Fig. 4(C)). Molecules 1252 and 8768, however, significantly reduced the number of activity bouts. Molecule 2060 decreased (*P* < 0.01) the volume of sucrose ingested (Fig. 4(D)).

When added to the blood meal, neither of the molecules significantly increased any of the feeding behaviors analyzed (Figs 5 and S1). Only the KR antagonist 8768 significantly reduced the number of sips (Fig. 5(A)). This molecule and 8796 also significantly (*P* < 0.01) reduced the volume of blood ingested (Fig. 5(D)). The high variability in the volume ingested with molecules 1252 and 8555 did not allow the detection of significant differences in ranks (Fig. 5(D)).

### 3.4 Effect of antagonist 8555 on feeding behaviors

**Figure 6:**
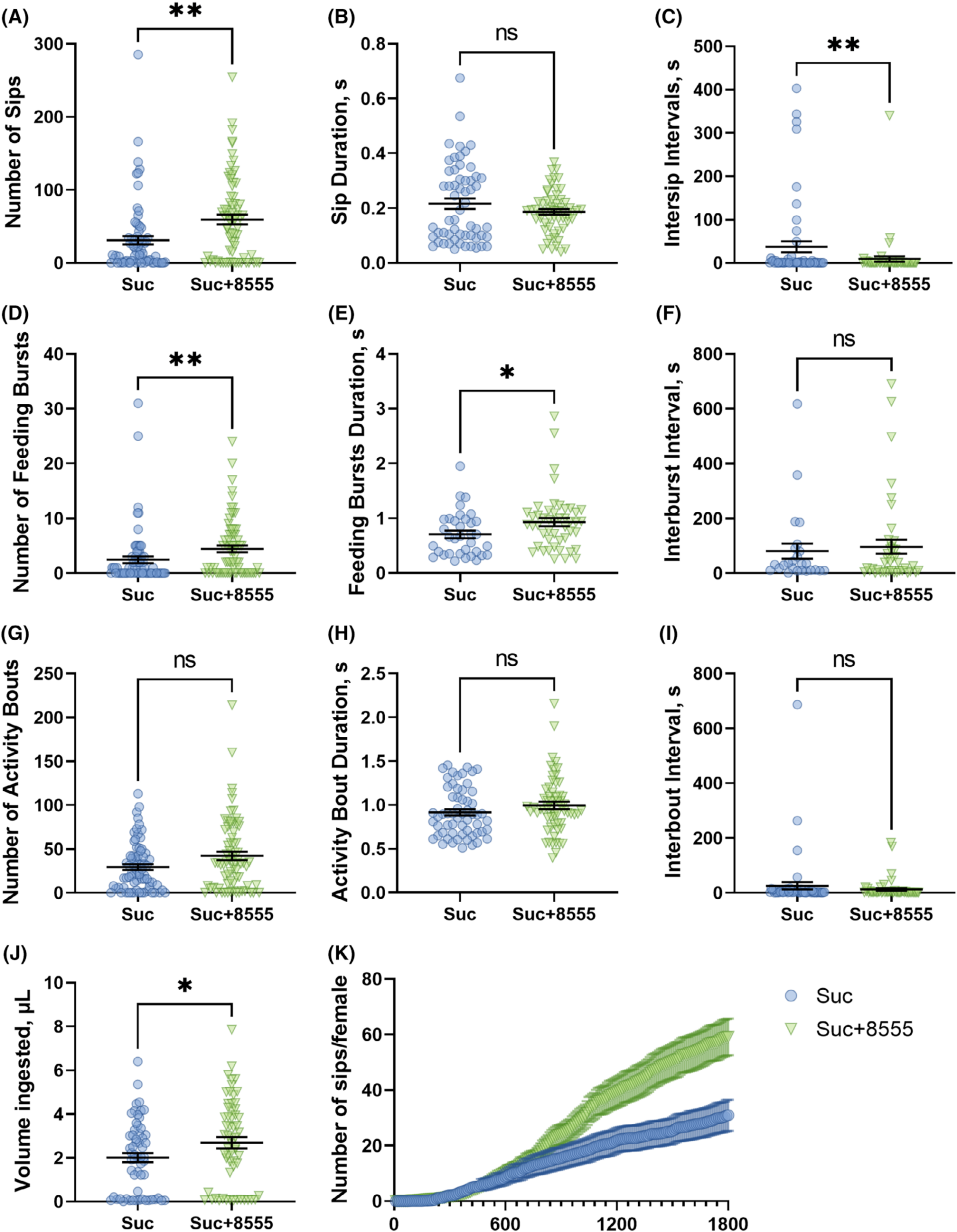
Comparison between feeding behaviors of female *Aedes aegypti* when offered 10% sucrose (circles in blue, Suc) or 10% sucrose + SACC‐0048555 at 1 mm (upside‐down triangles in green, Suc + 8555) using the flyPAD system in non‐choice assays. (A) Number of sips. (B) Duration of the sips (s). (C) Intersip intervals (s). (D) Number of feeding bursts, each characterized as three or more consecutive sips. (E) Duration of each feeding burst (s). (F) Duration of Interburst intervals (s). (G) Number of activity bouts, indicating how often the mosquito approaches the food. (H) Duration of the activity bouts (s). (I) Duration of interbout intervals (s). (J) Total volume ingested by each female. (K) Cumulative feeding, indicating the cumulative number of sips per female at every 10 s interval. Symbols represent outputs from individual mosquitoes, lines are means (*n* per treatment = 72) ± standard error of the mean (SEM). Mann–Whitney test, asterisks denote a statistical significance, where one asterisk (*) indicates *P* < 0.05, and two asterisks (**) indicate *P* < 0.01, and not significant (ns) indicates *P* > 0.05.

Figure 6 details each of the feeding behaviors analyzed using the flyPAD in non‐choice assays. The addition of molecule 8555 significantly increased the number of sips (59 ± 6.6) *versus* the control (31 ± 5.6) (Fig. 6(A)), the number of feeding bursts (4.4 ± 0.62) *versus* the control (2.4 ± 0.61) (Fig. 6(D)), and the volume ingested (2.7 ± 0.26) *versus* the control (2.0 ± 0.21) (Fig. 6(J)). The enhanced feeding is reflected in the kinetics of the cumulative number of sips per female throughout the 30 min assay duration (Fig. 6(K)). Mosquitoes offered sucrose containing 8555 also performed longer feeding bursts, spending on average 0.93 ± 0.076 s per burst compared to 0.70 ± 0.068 s in the control group (Fig. 6(E)), and had shorter intersip intervals, with a mean of 9.2 ± 6.3 s compared to the control 37 ± 13 s (Fig. 6(C)). However, there were no significant differences in the sip durations (Fig. 6(B)), activity bout durations (Fig. 6(H)), interburst intervals (Fig. 6(F)), or interbout intervals (Fig. 6(I)). This stimulation of feeding by KR antagonists is congruent with the sucrose antifeedant effect observed for the kinin agonist analog 1728.^68^ This peptidomimetic addition to the sugar meal induced aversive behaviors (fly‐, jump‐ or walk‐away), caused by direct inhibition of the sugar sensory neuron.^68^ Such duality mirrors neuropeptide‐Y (NPY) receptor pharmacology, where agonists suppress and antagonists stimulate feeding.^29^

Interestingly, despite the structural similarity among molecules within the quinoline family (Fig. 2), to which molecule 8555 belongs, the other analogs did not induce significant differences in feeding behavior. This difference may be attributed to the geometry of compound 8555, which is specified as the Z‐isomer (zusammen). In contrast, for the other molecules within this family, the stereochemistry at that position is undefined or unspecified, indicating that they may exist as a racemic mixture of stereoisomers. In bis‐quinoline scaffolds, the *E/Z* geometry can strongly influence molecular shape, π–π stacking interactions, and binding orientation, all of which may ultimately impact biological activity.

**Figure 7:**
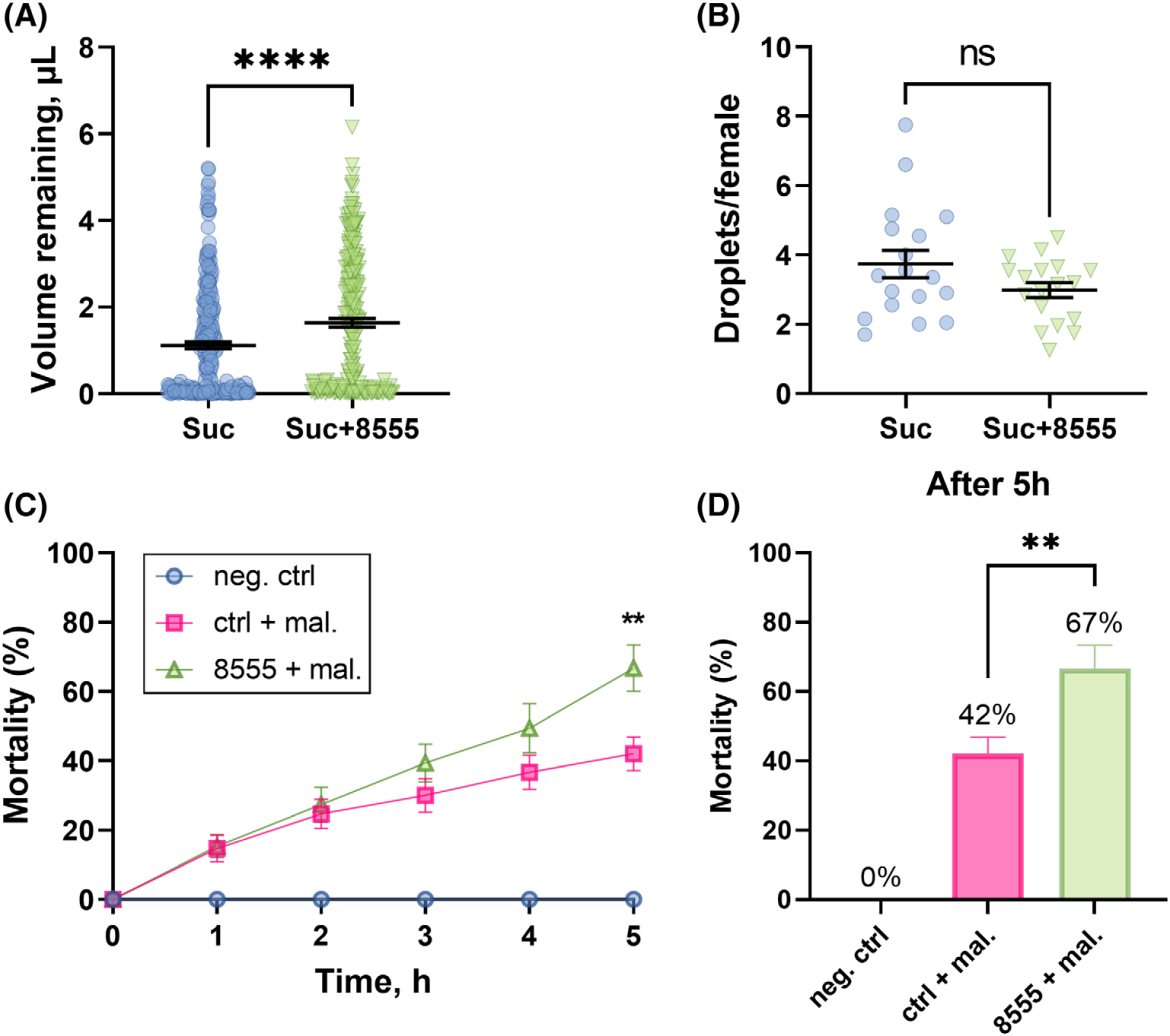
The effect of 10% sucrose supplemented with SACC‐0048555 on the remaining ingested meal volume, number of urine droplets excreted, and malathion efficacy. (A) Remaining meal volume in *Aedes aegypti* females after 5 h of exposure to the meals: sucrose (circles in blue, Suc) or sucrose mixed with SACC‐0048555 at 1 mm (upside‐down green triangles, Suc + 8555). Symbols represent individual females (*n* = 260 for each) and lines are mean ± standard error of the mean (SEM). (B) The average number of urine droplets excreted per female when exposed to control sucrose solution (Suc) or to sucrose solution containing SACC‐0048555 (1 mm; Suc + 8555) after 5 h of exposure. Horizontal lines represent mean ± SEM of the number of urine droplets per female obtained from 18 plates containing 20 females each. Symbols represent the average number of droplets per female. Mann–Whitney test, not significant (ns) indicates *P* > 0.05. (C) Mortality induced by contact with or ingestion of 0.66% malathion in 10% sucrose (magenta squares, ‘ctrl + mal.’), 0.66% malathion in 10% sucrose containing molecule SACC‐0048555 at 1 mm (green upside‐down triangles, ‘8555 + mal.’), or 10% sucrose alone (blue circles, ‘ctrl’). (D) Mortality induced by 0.66% malathion in 10% sucrose (magenta), 0.66% malathion in 10% sucrose containing molecule SACC‐0048555 at 1 mm (green) at the endpoint (5 h). For (C) and (D), symbols and bars are mean mortality. For each time point each treatment was adjudicated one plate, each with ten females, that is, *n* = 50 per treatment. Unpaired *t*‐test, asterisks denote a statistical significance, where two asterisks (**) indicate *P* < 0.01.

Aside from stimulating hindgut contractions, in mosquitoes the KR regulates chloride and fluid flux toward the Malpighian tubule lumen for fluid production and urine excretion.^14^, ^15^, ^69^ Since 8555 reduced hindgut contractions *ex vivo* (Fig. 3) and increased sucrose ingestion *in vivo* (Fig. 6), as expected for a KR antagonist, we hypothesized that (i) the ingestion of this molecule would result in reduced kinin‐induced fluid excretion, and (ii) this molecule could be used in the development of toxic sugar baits (TSB). Therefore, females starved for 24 h were placed in Petri dishes containing a drop of either sucrose or sucrose with 8555. After 5 h of *ad libitum* contact with the meals, volume ingested and urine droplets were counted (Fig. 7). The addition of 8555 resulted in a higher volume of sucrose remaining in the females, 1.6 ± 0.1 μL, compared to the control, 1.1 ± 0.1 μL (Fig. 7(A)). However, there were no significant differences in the mean number of excreted droplets of 3.7 ± 0.4 in the control and 3.0 ± 0.2 for 8555 (Fig. 7(B)). The fact that females ingested a larger volume of the solution with 8555 without a proportionate correlated increase in urine droplets indicated a reduction in excretion. Supporting this inference is that sucrose solution supplemented with 0.66% malathion in the presence of molecule 8555 increased female mortality by approximately 25% at the endpoint of 5 h (Fig. *7*(C),(D)).

Feeding modulators can significantly influence mosquito population dynamics by altering both survival and reproductive output. For example, attractive toxic sugar baits (ATSBs) exploit mosquitoes’ natural sugar‐feeding behavior to deliver oral toxins, achieving substantial reductions in adult mosquito densities in field trials.^70^ Insecticide‐laced baits represent a relatively new control strategy that is grounded in mosquito feeding ecology.^71^ In contrast, chemical repellents such as DEET primarily reduce blood‐feeding success by disrupting olfactory and gustatory pathways, thereby lowering host contact and pathogen transmission without necessarily decreasing overall mosquito abundance.^72^, ^73^, ^74^ Together, these approaches demonstrate that targeting feeding behavior represents a possible successful strategy for integrated mosquito population and disease control.^70^, ^75^

### 3.5 Evaluation of adulticidal activity by topical application and tarsal contact

During the second phase of the testing pipeline, all 88 molecules were applied topically onto *Ae. aegypti* and *Cx. quinquefasciatus* females to evaluate lethality (Supporting Information S4 Table S4). The 88 molecules were evaluated for mosquitocidal activity by initially applying them at a high concentration (1.25 mm) in acetone as carrier, onto females of *Ae. aegypti* and *Cx. quinquefasciatus*. From this initial screen, two compounds with potential for mosquito control were identified (Supporting Information S4 Table S4), SACC‐0039590 and SACC‐0428788, hereafter 9590 and 8788. Topical application of molecule 9590 resulted in a mortality of 89% ± 0.02% for *Ae. aegypti* and of 65% ± 0.06% for *Cx. quinquefasciatus* (Supporting Information S4 Table S4). Application of molecule 8788 resulted in a mortality of 54% ± 7% for *Ae. aegypti* and of 54% ± 8% in *Cx. quinquefasciatus* (Supporting Information S4 Table S4).

**Figure 8:**
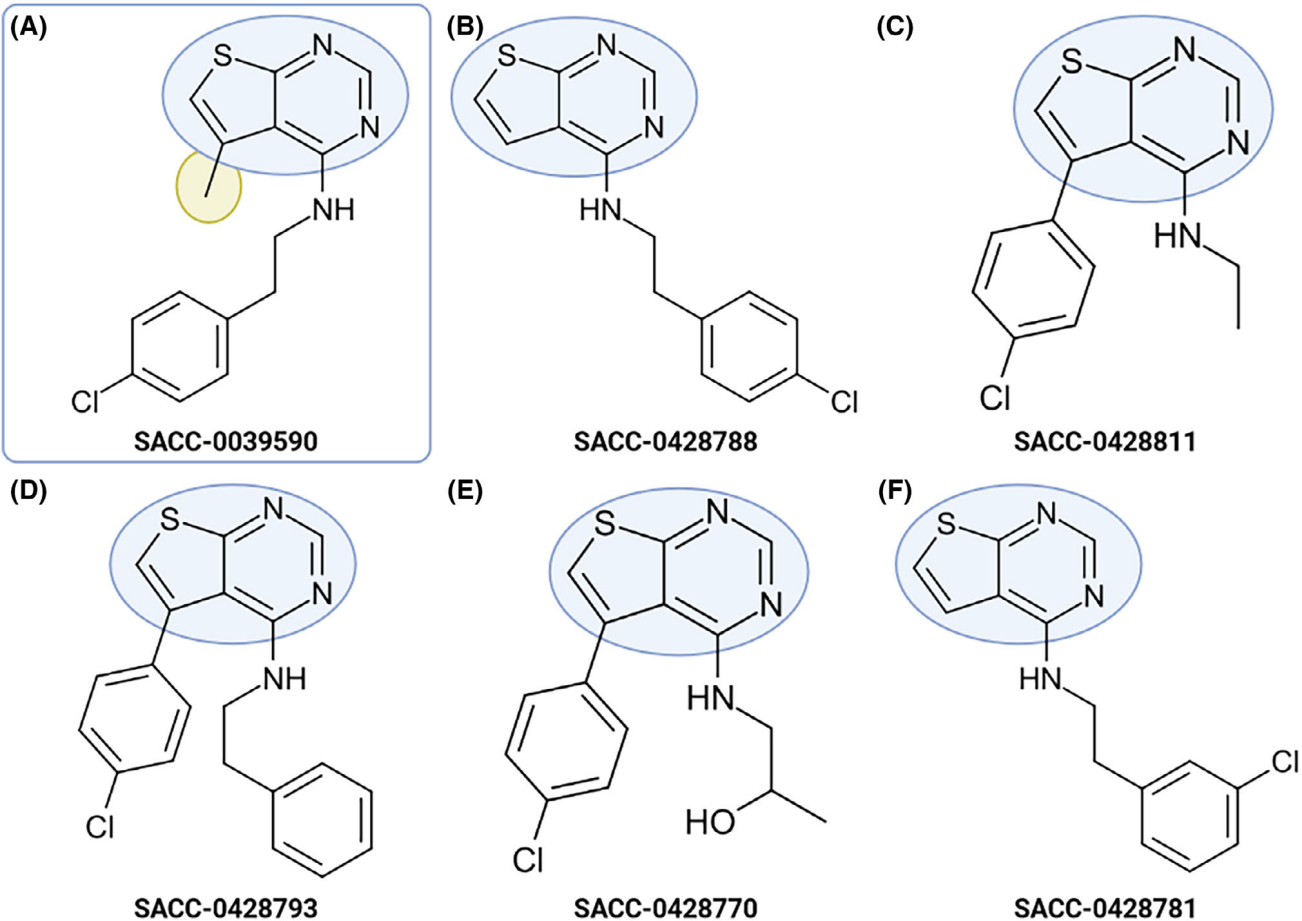
Structural similarity within the cluster derived from the ‘parent’ mosquitocidal molecule SACC‐0039590 (A).^30^ Structural analogs (B–F).^39^ The thieno[2,3‐d]pyrimidin groups are highlighted in blue and highlighted in yellow is the methyl group that differentiates the two mosquitocidal molecules SACC‐0039590 (A) and SACC‐0428788 (B). Created with BioRender.com

Although molecule 9590 was identified as an antagonist of the recombinant tick KR,^30^ it did not antagonize the recombinant *Ae. aegypti* KR (Supporting Information S4 Table 3). The application of either molecule, 9590 or 8788, did not inhibit the calcium fluorescence response elicited by the kinin agonist analog 1728 on IGKN‐G12 cells. Molecule 8788 is a structural analog of 9590, belonging to the same structure cluster (Fig. 8).^39^ Although 9590 was not cytotoxic to HDF,^30^ both molecules 9590 and 8788 have been described as cytotoxic against leukemia cells and osteoclasts.^76^ Comparably to our results, removing the methyl group (as in 8788) reduced the potency against these cells. Even though the similarities within the cluster range from 89% to 92%, only 9590 (Fig. 8(A)) and 8788 (Fig. 8(B)) caused significant mortality. This is likely because even subtle alterations in chemical structures may result in significant changes in binding affinity.

Molecules 9590 (*N*‐[2‐(4‐chlorophenyl)ethyl]‐5‐methylthieno[2,3‐d]pyrimidin‐4‐amine) and 8788 (*N*‐[2‐(4‐chlorophenyl)ethyl]thieno[2,3‐d]pyrimidin‐4‐amine) are thieno[2,3‐d]pyrimidines which contain a six‐membered pyrimidine ring fused to a five‐membered thiophene ring, and are structural analogs of purines. The exact structure of molecule 9590 has previously been cited in expired patents as inhibitors of histamine receptors^77^ and potassium channels,^78^ both for human medicinal purposes. Molecule 8788 had been covered by a now‐lapsed patent for controlling agricultural arthropod and fungal pests.^79^

Thienopyrimidine derivatives have been linked with various biological activities, including antimicrobial, anti‐inflammatory, analgesic, and anticancer through the inhibition of multiple enzymes and pathways.^80^, ^81^, ^82^ Numerous patents have been filed for thieno[2,3‐d]pyrimidine derivatives, identifying them as phosphodiesterase inhibitors and receptor antagonists, such as the metabotropic glutamate receptors, which are GPCRs that modulate synaptic transmission and neuronal excitability.^83^ These derivatives, along with thieno[3,2‐d]pyrimidine derivatives exhibit significant antitumor and radioprotective properties. Additionally, compounds based on thieno[2,3‐d]pyrimidine derivatives have been developed as immunomodulators and for the prevention and treatment of various diseases such as cerebral ischemia, malaria, tuberculosis, Alzheimer's, and Parkinson's disease. For extensive reviews, see Litvinov and Ali *et al*.^84^, ^85^ Additionally, this class of compounds has also shown promise as insecticides.^86^, ^87^, ^88^

Structural variation among the thienopyrimidine family (Fig. 8) occurs primarily in two sites, a substitution on the heterocyclic core and modifications to the amine‐linked side chain. As previously mentioned, molecule 9590 (Fig. 8(A)) differs from 8788 (Fig. 8(B)) only by the presence of a methyl substituent on the thienopyrimidine core. In contrast, molecules 8811 (Fig. 8(C)), 8793 (Fig. 8(D)), and 8770 (Fig. 8(E)) introduce the chlorobenzyl as a direct substituent on the thienopyrimidine core at the position occupied by a methyl group in 9590 (Fig. 8(A)) and left unsubstituted in 8788 (Fig. 8(B)). The amine side chain (Fig. 8(C)–(E)) further modulates size and polarity, ranging from small alkyl groups to larger aromatic extensions or hydroxylated chains. Finally, 8781 (Fig. 8(F)) retains the phenethylamine extension observed in 8788 (Fig. 8(B)), but they are positional isomers, differing only in the position of the chlorine substituent on the aromatic ring, corresponding to *para*‐ and *meta*‐chlorophenethyl analogs, respectively. Together, these structural changes alter steric bulk, flexibility, and potential intermolecular interactions, producing distinct SARs that help rationalize the observed variation in biological activity, including inactive analogs.

The new WHO standard operating procedure for testing the susceptibility of adult mosquitoes recommends mixing the active ingredient with a formulation of RME, a commercially available wetting agent, to increase solubility and enhance uptake of the insecticide by the mosquitoes.^89^ Vegetable oil‐based surfactants like RME are derived from seed or crop oils through processes like esterification or saponification. These non‐ionic surfactants are widely used in agriculture to enhance the effectiveness of pesticides by aiding in solubilizing, suspending, and dispersing active ingredients or enhancing the permeability through the insect exoskeleton.^90^, ^91^ Therefore, as a second step of the testing cascade, the ten antagonists of the mosquito KR were solubilized in acetone and re‐tested topically with the addition of RME. However, the addition of this adjuvant did not improve the lethality of any of the antagonistic molecules (Table 1). In *Ae. aegypti*, for the mosquitocidal 9590 the presence of adjuvant (RME) increased mortality rates to 94% ± 2% at 1 mm (Table 1) compared to 89% ± 2% when delivered at 1.25 mm in acetone only (Supporting Information S4 Table S4). Similarly, methylated vegetable oils reduced the lethal concentration required for different active ingredients against malaria vectors.^90^, ^92^, ^93^, ^94^, ^95^ Application of the molecule 8788 with the addition of the RME adjuvant significantly increased (*t*‐test, *P* < 0.01) mortality to 90% ± 2% (Table 1) compared to its application in acetone (54% ± 7%, Supporting Information S4 Table S4). In stark contrast, for *Cx. quinquefasciatus*, mortality levels were significantly reduced when females were treated topically with 9590 in RME (from 81% ±4% to 31% ± 3%, *t*‐test *P* < 0.0001). Also in contrast to results with *Ae. aegypti*, the addition of the RME to molecule 8788 significantly reduced (*t*‐test, *P* < 0.0001) its insecticidal activity, with *Cx. quinquefasciatus* female mortality reaching only 3% ± 1% (Table 1), compared to 54% ± 8% in acetone only.

**Table 1:** Topical application of selected antagonists of the recombinant *Aedes aegypti* kinin receptor

Molecule	*Aedes aegypti*		*Culex quinquefasciatus*	
	Mean	SEM	Mean	SEM
1252 1 mm	0%	0%	0%	0%
2060 1 mm	0%	0%	0%	0%
8555 1 mm	0%	0%	0%	0%
8618 1 mm	3%	2%	0%	0%
8768 1 mm	0%	0%	0%	0%
8771 1 mm	0%	0%	2%	2%
8773 1 mm	0%	0%	0%	0%
8774 1 mm	0%	0%	0%	0%
8775 1 mm	0%	0%	0%	0%
8796 1 mm	0%	0%	0%	0%
9590 1 mm	94%	2%	31%	3%
8788 1 mM	90%	2%	3%	1%
Negative control	0%	0%	0%	0%

Molecules (0.2 μL) were applied at 1 mm in acetone containing rapeseed oil methyl esters at 0.392 mg/mL on the dorsal thorax of 3–5‐day‐old unfed females of *Ae. aegypti* Liverpool strain and *Cx. quinquefasciatus* Sebring strain. The solution concentration equates to a dose of 0.2 nmol per female. Percentages are the mean of three replicates of 20 females. SEM, standard error of the mean. The negative control consisted of the above carrier mixture only.

**Figure 9:**
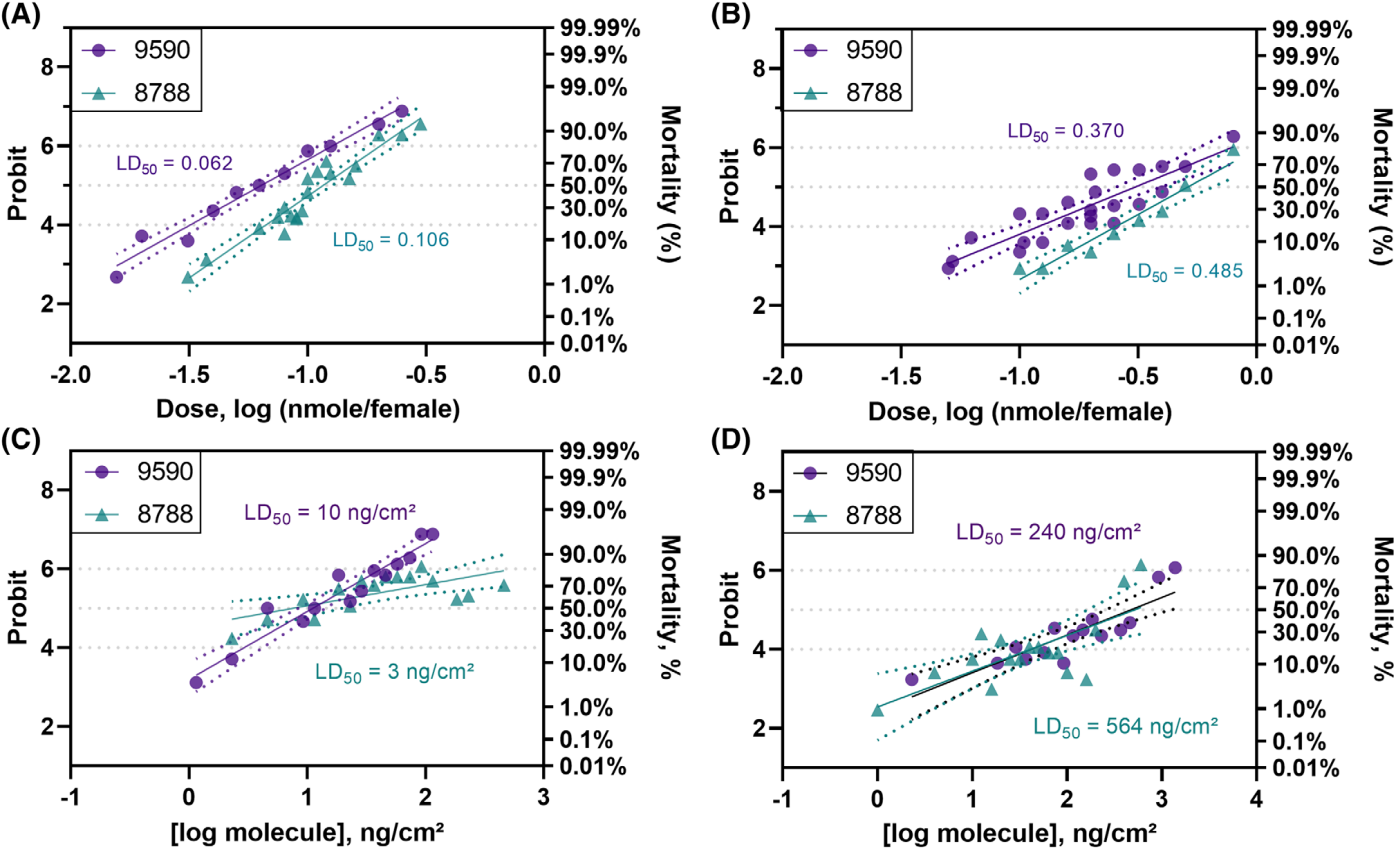
Probit analysis of thorax topical application (A and B) or vial (C and D) bioassays of female *Aedes aegypti* (A and C), Liverpool strain, and *Culex quinquefasciatus* , Sebring strain, (B and D) for molecules SACC‐0039590 (circles in purple) and SACC‐0428788 (triangles in teal). Serial dilutions were prepared in acetone containing rapeseed oil methyl esters (RMEs) at 0.392 mg/mL for topical applications and 0.098 mg/mL or 0.049 mg/mL for vial assays with *Ae. aegypti* or *Cx. quinquefasciatus*, respectively. For topical applications, symbols represent 90 mosquitoes for *Ae. aegypti* and 60 mosquitoes for *Cx. quinquefasciatus*. For vial assays, symbols represent 30 mosquitoes for either species. Dotted lines are 95% confidence intervals.

The two mosquitocidal molecules, 9590 and 8788, were then evaluated in dose–response bioassays (Fig. 9). Probit analyses of the dose‐mortality data obtained when molecule concentrations from 0.078 to 1.5 mm in RME applied to the *Ae. aegypti* thorax yielded an LD_50_ of 0.062 nmole/female for 9590 and 0.106 nmole/female for 8788 (Fig. 9(A)), which equates to 18.8 and 30.7 ng/female, respectively. For *Cx. quinquefasciatus* dilutions ranged from 0.13 to 4 mm and the LD_50_ was 0.37 nmole/female for 9590 and 0.485 nmole/female for 8788 (Fig. 9(B)), equating to 112.4 and 140.5 ng/female respectively. These probit analyses with RME revealed that the molecules kill at sub‐micromolar concentrations (Fig. 9). However, for *Ae. aegypti*, the molecule 9590 was more potent than for *Cx. quinquefasciatus* by a factor of six at the LD_50_ (Fig. 9(A),(B)), while molecule 8788 was more potent by a factor of five at the LD_50_ (Fig. 9(A),(B)).

Interestingly, adding to the potential applications of these compounds, molecules 9590 and 8788 also showed adulticidal activity upon tarsal contact when tested in vial bioassays (Figure 9(C),(D)). For vial assays with *Ae. aegypti*, probit analyses using solutions ranging between 200 and 1 μm resulted in LD_50_ of 10 and 3 ng/cm^2^ (Fig. *9*(C)), for molecules 9590 and 8788, respectively, corresponding to 0.83 and 0.25 μg/vial. For *Cx. quinquefasciatus*, probit analyses using solutions ranging between 600 and 1 μm resulted in LD_50_ of 240 and 564 ng/cm^2^ (Fig. 9(D)), for molecules 9590 and 8788, respectively. These dosages correspond to 20 and 47 μg/vial. Molecule 9590 was more potent against *Ae. aegypti* by a factor of 24 at the LD_50_ when compared to *Cx. quinquefasciatus*, and molecule 8788 was more potent for *Ae. aegypti* by a factor of 188 at the LD_50_.

**Figure 10:**
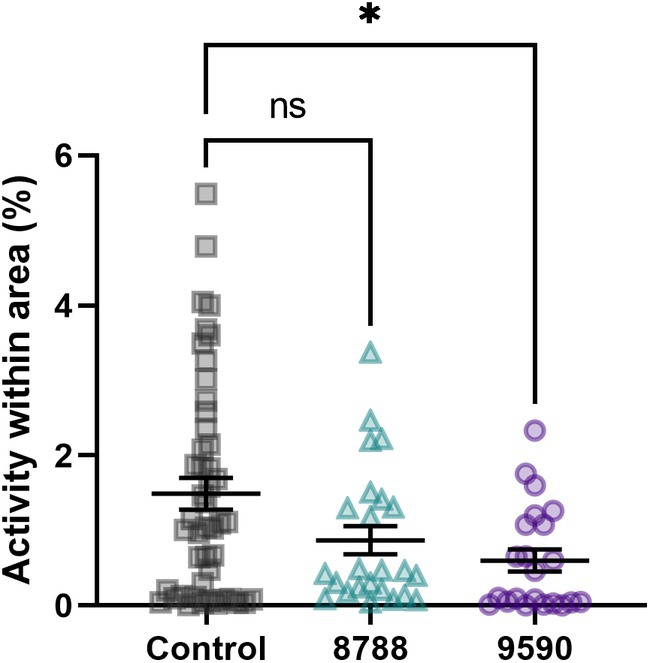
Assessment of mosquitocidal molecules as inhibitors of hindgut contraction *ex vivo*. Isolated hindguts from non‐blood fed females of *Aedes aegypti* were preincubated with antagonists (100 μm) or saline control for 5 min. Subsequently, the kinin agonist analog 1728 ([Aib]FF[Aib]WGamide) (10 μm) was added. Tissues were filmed for 30 s at 1 h post‐treatment and analyzed using Ethovision XT 17. The control consisted of application of agonist analog only, and molecules are represented by the last four digits of their identifier, namely SACC‐0428788 (triangles in teal) and SACC‐0039590 (circles in purple), respectively. Symbols represent activity of individual hindguts and lines are means (*n* = 47 for control and 24 for each molecule) ± standard error of the mean (SEM). Kruskal–Wallis followed by Dunn's multiple comparisons test, asterisks denote statistical significance, where three asterisks (***) indicate *P* < 0.05, and not significant (ns) indicates *P* > 0.05.

Beyond lethality, although these molecules did not antagonize the recombinant *Ae. aegypti* KR (Supporting Information S4 Table S3), 9590 significantly reduced the hindgut activity when compared to the control tissues (Fig. 10, *P* < 0.05). There was no difference in ranks in the activity within area of tissues incubated with molecule 8788 (Fig. 10). This inhibition suggests that molecule 9590 reduced hindgut contractions independently of KR antagonism, indicating the presence of a target in the hindgut and/or tissues associated with it (e.g., nerves, muscles). Despite the lethality, the mode of action of the two mosquitocidal molecules is still unknown.

### 3.6 Effect of sublethal concentrations of mosquitocidal molecules on *Ae. aegypti* temperature resistance and female behavior

**Figure 11:**
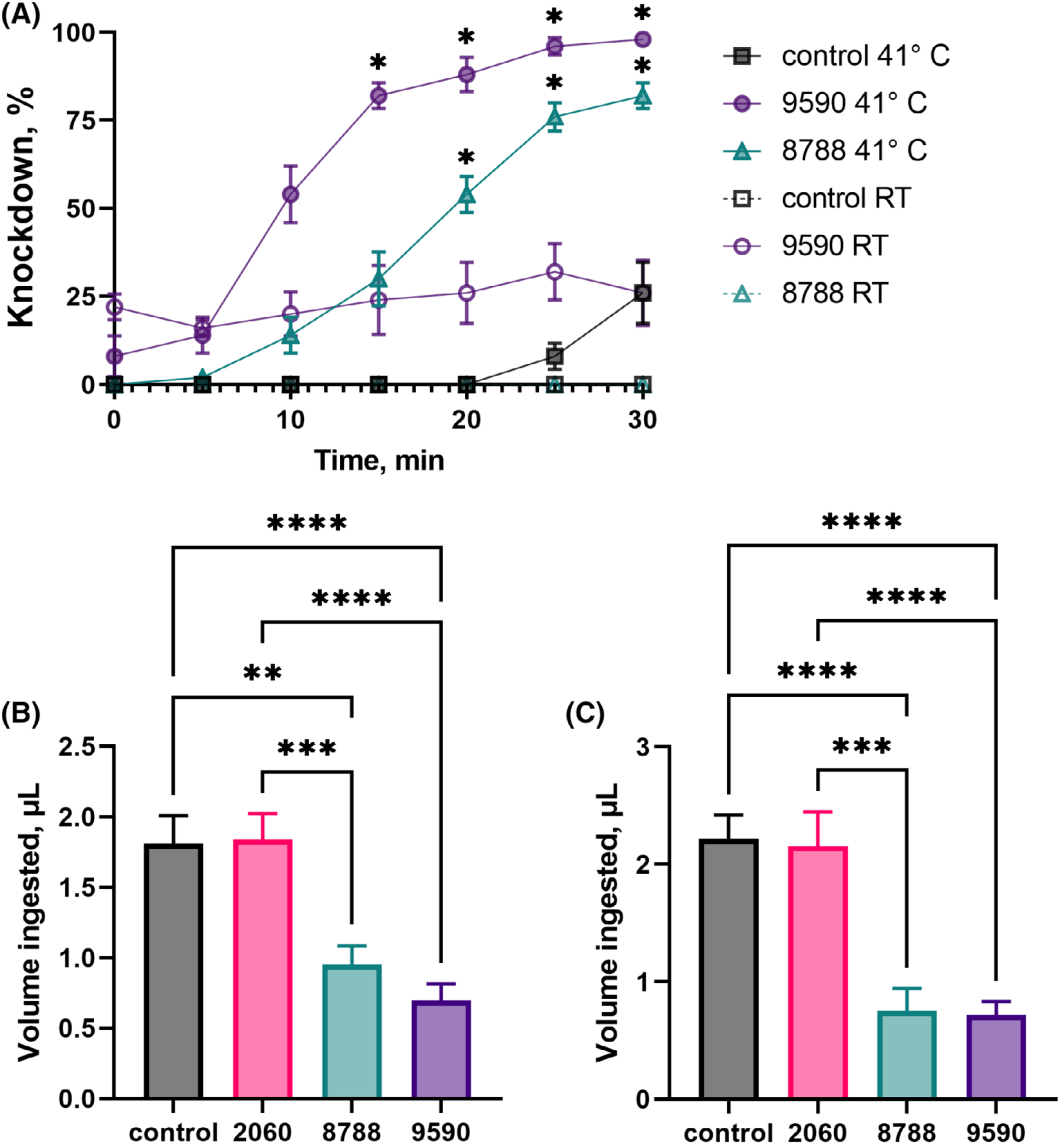
Evaluation of the effect of mosquitocidal molecules at sublethal concentrations (LC_25_) on *Aedes aegypti* temperature resistance and blood‐feeding. (A) Effect of spraying solution of molecule SACC‐0428788 or SACC‐0039590 at the LC_25_ (0.2 mm) on high temperature resistance. Mosquitoes treated with the molecules were significantly knocked down by increased temperature (41 °C) in comparison to the controls and mosquitoes kept at room temperature (RT). Symbols are mean percentage of knocked down mosquitoes ± standard error of the mean (SEM) (three series of five replicates with ten females each). Repeated measures two‐way ANOVA. To assess effects on blood‐feeding, females were treated topically on the thorax with 0.2 μL of either solvent only (0.392 mg/mL RME in acetone) or molecules SACC‐0412060, SACC‐0428788 or SACC‐0039590 (0.2 mm in the same solvent) using a syringe and a repeating dispenser (B) or sprayed with 0.2 mm solutions using a handheld atomizer (C). Mosquitoes were allowed to recover for 1 h and offered blood for 30 min. At the end point, mosquitoes were collected, and the volume ingested was estimated (see Materials and Methods for details). Histograms show means ± standard error of the mean (SEM) per female. For (A), the meal volume was estimated from 216 females per treatment, and replicates were of 24 mosquitoes per group. For (B), 255 females were in the control group, 115 were treated with 2060, 124 were treated with 8788, and 255 females with 9590. One‐way ANOVA, followed by Tukey's multiple comparisons test. Asterisks denote statistical significance, where one asterisk (*) indicates *P* < 0.05, two asterisks (**) indicate *P* < 0.01, three asterisks (***) indicate *P* < 0.001, and four asterisks (****) indicate *P* < 0.0001.

Arbovirus transmission control relies on vector control, host detection interference, or blood‐feeding disruption.^96^ Therefore, after observing the lethality of molecules 9590 and 8788 against *Ae. aegypti*, the effects of these molecules on different physiological processes, such as high temperature tolerance, blood‐feeding behavior, and meal ingestion were assessed. To assess the impact of high temperature, females sprayed with 9590 or 8788 at the sublethal dose of LC_25_ (0.2 mm) were exposed to 41 °C (Fig. 11(A)). For females treated with 9590, although there was an initial knockdown of approximately 8% caused by the molecule itself, 98% of females were unresponsive after 30 min. This represented a statistically significant difference (~72%) in knockdown compared to the control group (Fig. 11(A)). Similarly, approximately 82% of females sprayed with 8788 and kept at 41 °C were knocked down after 30 min (Fig. 11(A)). In contrast, at RT, only approximately 26% of females sprayed with 9590 were knocked down, and no knockdown effect was observed in the control group or in females sprayed with 8788 within the 30 min of observation. Susceptibility to high temperatures may be a result of changes in metabolism, binding‐affinity and/or chemical uptake generated by the temperature‐related stress. All of which can influence their metabolism, and the ability to detoxify insecticides.^97^ Similarly, ambient temperature can directly affect the susceptibility of these mosquitoes to pyrethroids.^97^, ^98^

To evaluate the blood‐feeding capability of surviving mosquitoes, females were topically treated with either solvent only, 9590, 8788, or 2060, the latter was chosen as a negative control because it was a non‐lethal KR antagonist (Table 1 and Fig. 1), all at 0.2 mm, the LC_25_ for 9590 (Fig. 11(B),(C)). At sublethal doses, molecules impaired the mosquitoes’ blood‐feeding capability, as surviving females treated with mosquitocidal molecules ingested a significantly smaller meal volume than the solvent control and females treated with 2060 (Fig. 11(B)). While these two latter groups ingested 1.8 ± 0.2 μL each, females treated with either 9590 or 8788 ingested less (0.95 ± 0.14 μL, and 0.70 ± 0.12 μL, respectively) (Fig. 11(B)). To evaluate field application feasibility, molecules were sprayed using a small atomizer (Fig. 11(C)), and this method yielded similar results to the topical assay. Females sprayed with 9590 ingested 0.72 ± 0.12 μL and females sprayed with 8788 ingested 0.75 ± 0.19 μL, that is, significantly smaller meal sizes compared to the control (2.2 ± 0.2 μL) and females sprayed with 2060 (2.2 ± 0.3 μL) (Fig. 11(C)).

**Figure 12:**
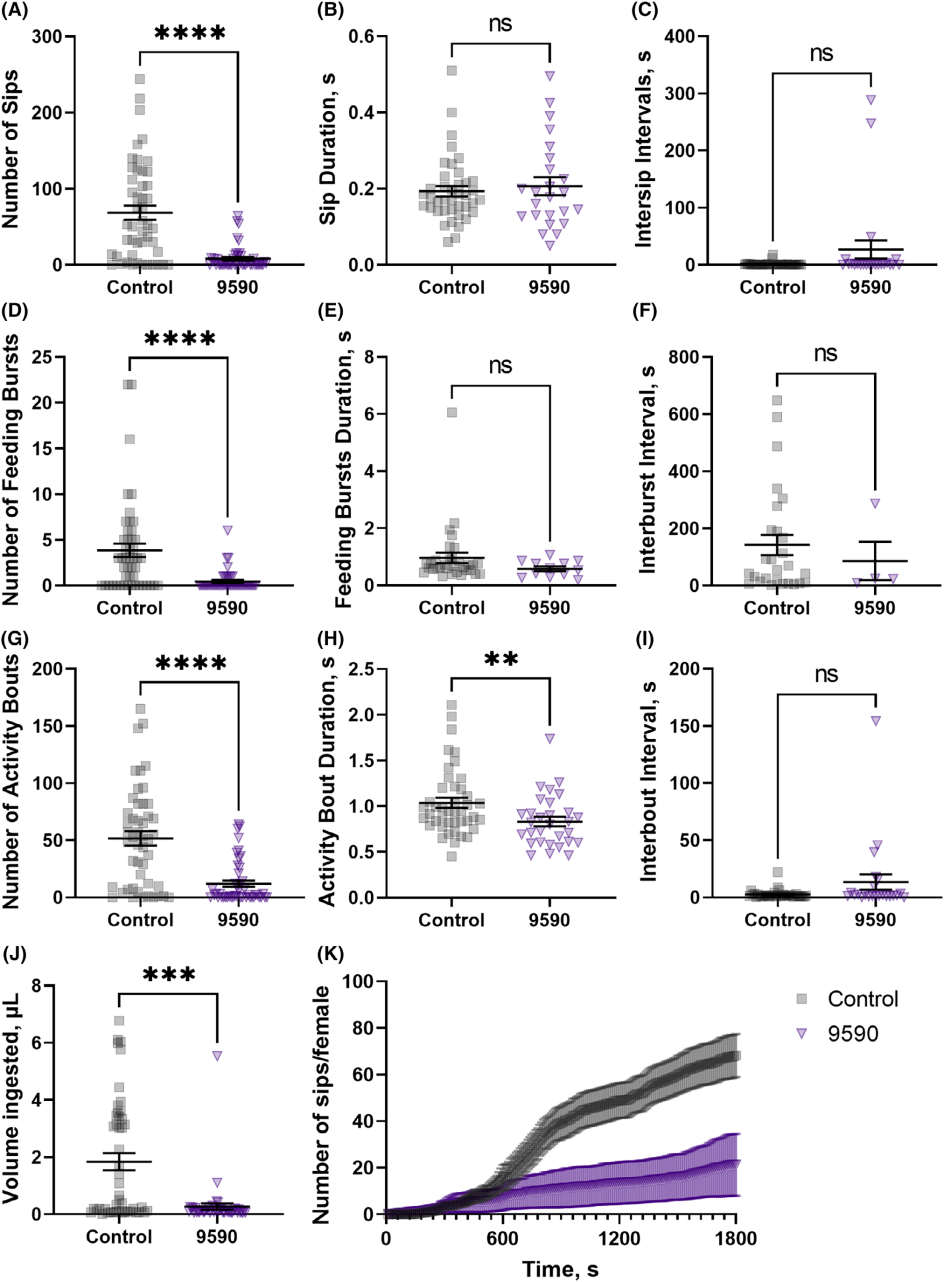
Effect of the atomizer‐spray application of SACC‐0039590 at a sublethal dose on the blood‐feeding behaviors of females of *Aedes aegypti* assessed by the flyPAD system. Control females (squares in black) were sprayed with solvent only (0.392 mg/mL RME in acetone) and treated females (upside‐down triangles in purple, 9590) were sprayed with a 0.2 mm solution of SACC‐0039590. Females were allowed 1 h recovery before being anesthetized and placed in the flyPAD. (A) Number of sips. (B) Duration of the sips (s). (C) Intersip intervals (s). (D) Number of feeding bursts, each characterized as three or more consecutive sips. (E) Duration of each feeding burst (s). (F) Duration of interburst intervals (s). (G) Number of activity bouts, indicating how often the mosquito approaches the food. (H) Duration of the activity bouts (s). (I) Duration of interbout intervals (s). (J) Total volume ingested by each female. (K) Cumulative feeding, indicating the cumulative number of sips per female at 10 s intervals. Symbols represent outputs from individual mosquitoes, lines are means (*n* per treatment = 72) ± standard error of the mean (SEM). Mann–Whitney test, asterisks denote a statistical significance, where two asterisks (**) indicate *P* < 0.01, three asterisk (***) indicate *P* < 0.001, and four asterisks (****) indicate *P* < 0.0001, and not significant (ns) indicates *P* > 0.05.

**Figure 13:**
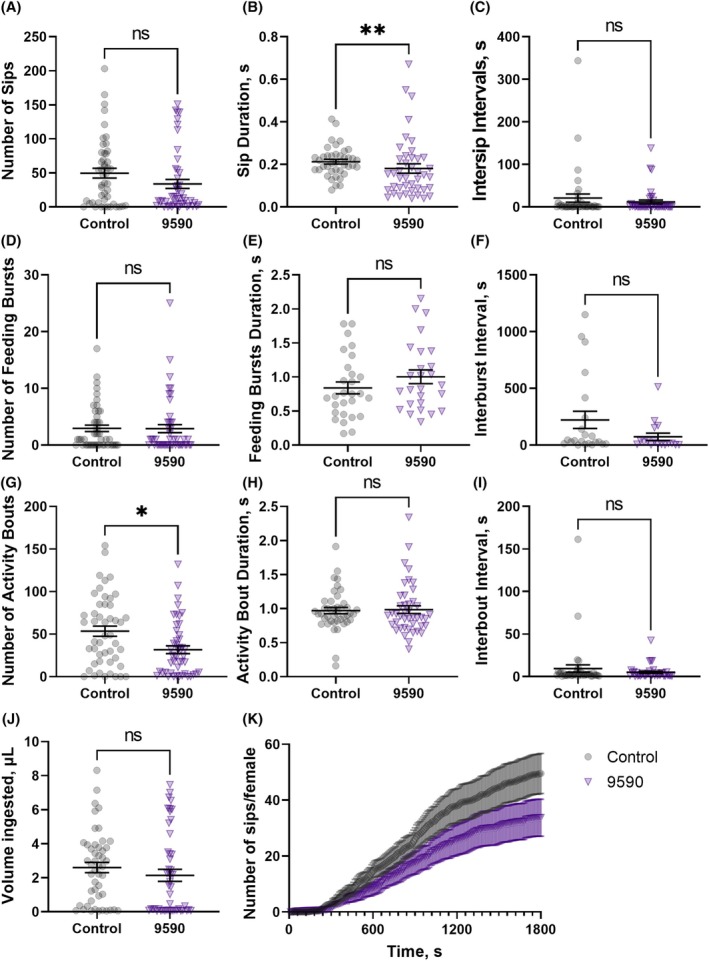
Effect of the spray application of SACC‐0039590 at a sublethal concentration using an atomizer on the sugar‐feeding behaviors of female *Aedes aegypti* assessed with the flyPAD system. Control females (squares in black) were sprayed with the solvent only (0.392 mg/mL RME in acetone) and treated females (upside‐down triangles in purple, 9590) were sprayed with a solution of SACC‐0039590 at 0.2 mm. Females were allowed 1 h recovery before being anesthetized and placed in flyPAD. (A) Number of sips. (B) Duration of the sips (s). (C) Intersip intervals (s). (D) Number of feeding bursts, each characterized as three or more consecutive sips. (E) Duration of each feeding burst (s). (F) Duration of interburst intervals (s). (G) Number of activity bouts, indicating how often the mosquito approaches the food. (H) Duration of the activity bouts (s). (I) Duration of interbout intervals (s). (J) Total volume ingested by each female. (K) Cumulative feeding, indicating the cumulative number of sips per female recorded every 10 s. Symbols represent outputs from individual mosquitoes, lines are means (*n* per treatment = 72) ± standard error of the mean (SEM). Mann–Whitney test, asterisks denote a statistical significance, where two asterisks (**) indicate *P* < 0.01, three asterisks (***) indicate *P* < 0.001, and four asterisks (****) indicate *P* < 0.0001, and not significant (ns) indicates *P* > 0.05.

Females sprayed with both molecules were evaluated for their blood‐feeding (Fig. 12) and sugar‐feeding behaviors (Fig. 13) using the flyPAD to determine if females would still probe the meals (recorded as ‘sips’) even if the feeding itself (volume ingestion) was impaired (Fig. 11). Mosquitoes sprayed with sublethal doses of 9590 sipped significantly less blood compared to the solvent‐only control group (Fig. 12(A)). Control females probed an average of 68 ± 9 times, while those sprayed with 9590 probed only 8 ± 2 times (Fig. 12(A)). The same was observed for the number of feeding bursts and feeding bouts. Sprayed females averaged 0.5 ± 2 feeding bursts and 12 ± 3 activity bouts, compared to 4 ± 0.7 and 52 ± 6, respectively, for the control mosquitoes (Fig. 12(A),(G), respectively). Additionally, the average activity bout duration (Fig. 12(H)) was shorter for the treated females (0.83 ± 0.05 s) compared to the control (1.0 ± 0.06 s). Altogether, differential behaviors resulted in a smaller blood meal ingested by the females sprayed with 9590 (0.3 ± 0.1 μL) than the control (1.8 ± 0.3 μL), (Fig. 12(J)). There were no significant differences in the sip duration (Fig. 12(B)), duration of intersip intervals (Fig. 12(C)), feeding burst durations (Fig. 12(E)), interburst intervals (Fig. 12(F)), or interbout intervals (Fig. 12(I)). Figure 12(K) summarizes the feeding kinetics as the cumulative number of sips or probing performed up to any time point, for 30 min. Therefore, surviving mosquitoes exposed to these molecules would, at least temporarily, be severely impacted by temperature stress and have impaired blood‐feeding competence.

Spraying females with 9590 (Fig. 13), did not affect the number of sips taken of sucrose solution (Fig. 13(A)) or feeding bursts (Fig. 13(D)). The molecule did not significantly alter the duration of feeding bursts (Fig. 13(E)) or activity bouts (Fig. 13(H)). It did not increase the intersip, interburst or interbout intervals (Fig. 13(C),(F),(I), respectively). However, females sprayed with 9590 at 0.2 mm performed significantly less activity bouts (Fig. 13(G)). They performed 32 ± 5 activity bouts compared to the control group which performed 53 ± 6. Treated females also took shorter sips, with average duration of 0.18 ± 0.02 s, compared to 0.21 ± 0.01 s in control females (Fig. 13(B)). However, the treatment did not significantly impact the volume ingested (Fig. 13(J)). Control females ingested 2.6 ± 0.3 μL of sucrose and females treated with 9590 ingested 2.1 ± 0.4 μL. Both groups were similar in the kinetics of the cumulative sips taken (Fig. 13(K)).

In a supplementary assay, when 9590 was added directly to the sucrose solution it did not significantly decrease the number of sips, bursts or bouts, but, in contrast to the spraying treatment, it did reduce the total volume ingested (Fig. S1, panel 20).

## 4 CONCLUSION

This study reported the discovery of novel bioactive chemistries on the *Ae. aegypti* KRs. Among 88 molecules evaluated, ten showed significant antagonistic activity against the mosquito KR. Molecule 8555 notably reduced hindgut contractions, stimulated sucrose feeding behavior, and increased ingestion of malathion leading to 25% more mortality, suggesting its potential use in developing feeding stimulants and ATSB. Altogether the effects of KR antagonists causing inhibition of mosquito hindgut activity, the stimulation of feeding, and the increase in meal volume remaining after ingesting an antagonist, agree with the known functions of the KR *in vivo* as stimulating hindgut contractions,^14^, ^15^, ^30^ inhibiting sucrose detection in labella and tarsi,^68^ and increasing diuresis.^14^, ^15^, ^99^, ^100^ Topical screening identified two mosquitocidal thieno[2,3‐d]pyrimidines molecules, 9590 and 8788, which at sublethal doses also significantly decreased blood‐feeding behavior and meal ingestion. These findings underscore the potential of small molecules as arthropod‐specific pharmacological tools for discovering novel aspects of mosquito behavior and insecticide development for vector control.

## FUNDING INFORMATION

This research was funded by the US Department of Defense, under the Deployed War‐Fighter Protection (DWFP) Program (Project Number W911QY2210005) to PVP, and partially by a seed grant in Insect Vector Diseases Program (FY ‘22‐‘23–‘24‐‘25) from Texas A&M AgriLife Research to PVP.

## CONFLICT OF INTEREST

A United States patent application Publication Number: US 2024/0251785 A1 has been filed Publication Date: 1 August 2024, P. V. Pietrantonio, Caixing Xiong and Dwight D. Baker. Small molecule antagonists and agonists of arthropod kinin receptors for pest control. A Provisional Patent Application is being prepared P14705US00. P. V. Pietrantonio, James C. Sacchettini, C. Xiong, and B. M. Henriques‐Santos. Small molecules as acaricidal kinin receptor antagonists or mosquitocidal agents.

## AUTHOR CONTRIBUTIONS

BMH‐S: design, methodology, investigation, formal analysis, data curation, visualization, writing original draft, and editing; PVP: conceptualization, design, resources, data curation, project administration, funding acquisition, investigation, writing, and editing final manuscript. All authors reviewed the final manuscript.

## Supporting information


**Figure S1.** FlyPAD assays with all antagonistic or mosquitocidal molecules in sucrose or blood. Comparison between feeding behaviors of female *Aedes aegypti* when offered control meals (blood or 10% sucrose) or the meal containing the test molecule at 1 mm (blood or suc + molecule number) using the flyPAD system in non‐choice assays. (A) Number of sips. (B) Duration of the sips (s). (C) Intersip intervals (s). (D) Number of feeding bursts, each characterized as three or more consecutive sips. (E) Duration of each feeding burst (s). (F) Duration of Interburst intervals (s). (G) Number of activity bouts, indicating how often the mosquito approaches the food. (H) Duration of the activity bouts (s). (I) Duration of interbout intervals (s). (J) Total volume ingested by each female. (K) Cumulative feeding, indicating the cumulative number of sips per female at every 10 s interval. Symbols represent outputs from individual mosquitoes, lines are mean ± standard error of the mean (SEM). Mann–Whitney test, asterisks denote a statistical significance, where one asterisk (*) indicates *P* < 0.05, and two asterisks (**) indicate *P* < 0.01, three asterisks (***) indicate *P* < 0.001, four asterisks (****) indicate *P* < 0.0001, and not significant (ns) indicates *P* > 0.05.

**Video S1.** Video of control mosquito hindgut treated with 10 μm of the kinin agonist analog 1728 ([Aib]FF[Aib]WGamide) showing induced contractions. Video recording of mosquito midgut contraction after 5 min of exposure. The video was analyzed in EthoVision XT 17 with activity analysis showing changes in activity (magenta highlights).

**Video S2.** Video of mosquito hindgut treated with 100 μm of the KR antagonistic small molecule SACC‐0048555 before the addition of 10 μm of kinin agonist analog 1728 ([Aib]FF[Aib]WGamide) showing the inhibition of the induced contractions. Video recording of tick midgut contraction after 5 min of exposure to antagonist and 5 min of exposure to kinin agonist. The video was analyzed in EthoVision XT 17 with activity analysis showing minimal changes in activity (magenta highlights). Hindgut was stained pink as a result of the incubation with molecule 8555, which has a purple coloration.

**Table S1.** Chemical identities of selected tick kinin receptor antagonists from^30^ (35 molecules) and 53 structural analogs.^39^ Molecules are listed based on their CDD vault identifier, CAS number, SMILES, vendor, and catalog number.
**Table S2.** Structural families of molecules based on the 11 drug‐like tick kinin antagonist hits^30^ and structures of the selected analogs based on at least 80% similarity, as calculated by SciFinder.^39^
**Table S3.** Characterization of 88 molecules in the dose–response assay. Dose–response curves were generated from the calcium fluorescence responses of the recombinant mosquito kinin receptor (IGKN‐G12) and vector‐only cell (V/O) in the dual‐addition assay (described under Materials and Methods section). The relative fluorescence units (RFU) in the *Y*‐axis were normalized by subtracting the background signal. Inhibition percentage was calculated based on the negative control injected with buffer only. Molecules highlighted in cyan are the parent molecules reported,^30^ and molecules highlighted in red are selected antagonists.
**Table S4.** Topical application of small molecules on the dorsal thorax of 3–5‐day‐old unfed females *Aedes aegypti* and *Culex quinquefasciatus* . Molecules (0.2 μL) were applied at 1.25 mm in acetone, which equates to 0.25 nmole/female. Negative control consisted of acetone only and positive control consisted of 0.1 mm Permethrin in acetone. Numbers are mean of three replicates of 20 females ± standard error of the mean (SEM), except for molecules SACC‐003959590 and SACC‐0428788, which were replicated six times. Two‐way ANOVA followed by Dunnett's multiple comparisons test. Superscript letter (a) denotes statistical significance (*P* > 0.0001).

## Data Availability

The data that support the findings of this study are available from the corresponding author upon reasonable request.
