# Supplementary material for: Small molecules as arthropod kinin receptor antagonists, feeding modulators, or novel mosquitocidal agents
Source: Pest Manag Sci. 2026 Apr 10;82(7):6842–63. doi: 10.1002/ps.70764 (PMC13240700; doi:10.1002/ps.70764)

**Figure S1**. FlyPAD assays with all antagonistic or mosquitocidal molecules in sucrose or blood. Comparison between feeding behaviors of female Aedes aegypti when offered control meals (blood or 10% sucrose) or the meal containing the test molecule at 1 mM (blood or suc + molecule number) using the flyPAD system in non-choice assays. **A.** Number of sips. **B.** Duration of the sips (s). **C.** Intersip intervals (s). **D.** Number of feeding bursts, each characterized as three or more consecutive sips. **E.** Duration of each feeding burst (s). **F.** Duration of Interburst intervals (s). **G.** Number of activity bouts, indicating how often the mosquito approaches the food. **H.** Duration of the activity bouts (s). **I.** Duration of interbout intervals (s). **J.** Total volume ingested by each female. **K.** Cumulative feeding, indicating the cumulative number of sips per female at every 10 s interval. Symbols represent outputs from individual mosquitoes, lines are means ± standard error of the mean (SEM). Mann-Whitney test, asterisks denote a statistical significance, where one asterisk (*) indicates P < 0.05, and two asterisks (**) indicate P < 0.01, three asterisks (***) indicate P < 0.001, four asterisks (****) indicate P < 0.0001, and not significant (ns) indicates P > 0.05.

Panel 1: Blood with molecule SACC-0121252. This partial antagonist of the *Ae. aegypti* kinin receptor did not alter any of the feeding variables analyzed by the flyPAD and did not affect the meal volume ingested.
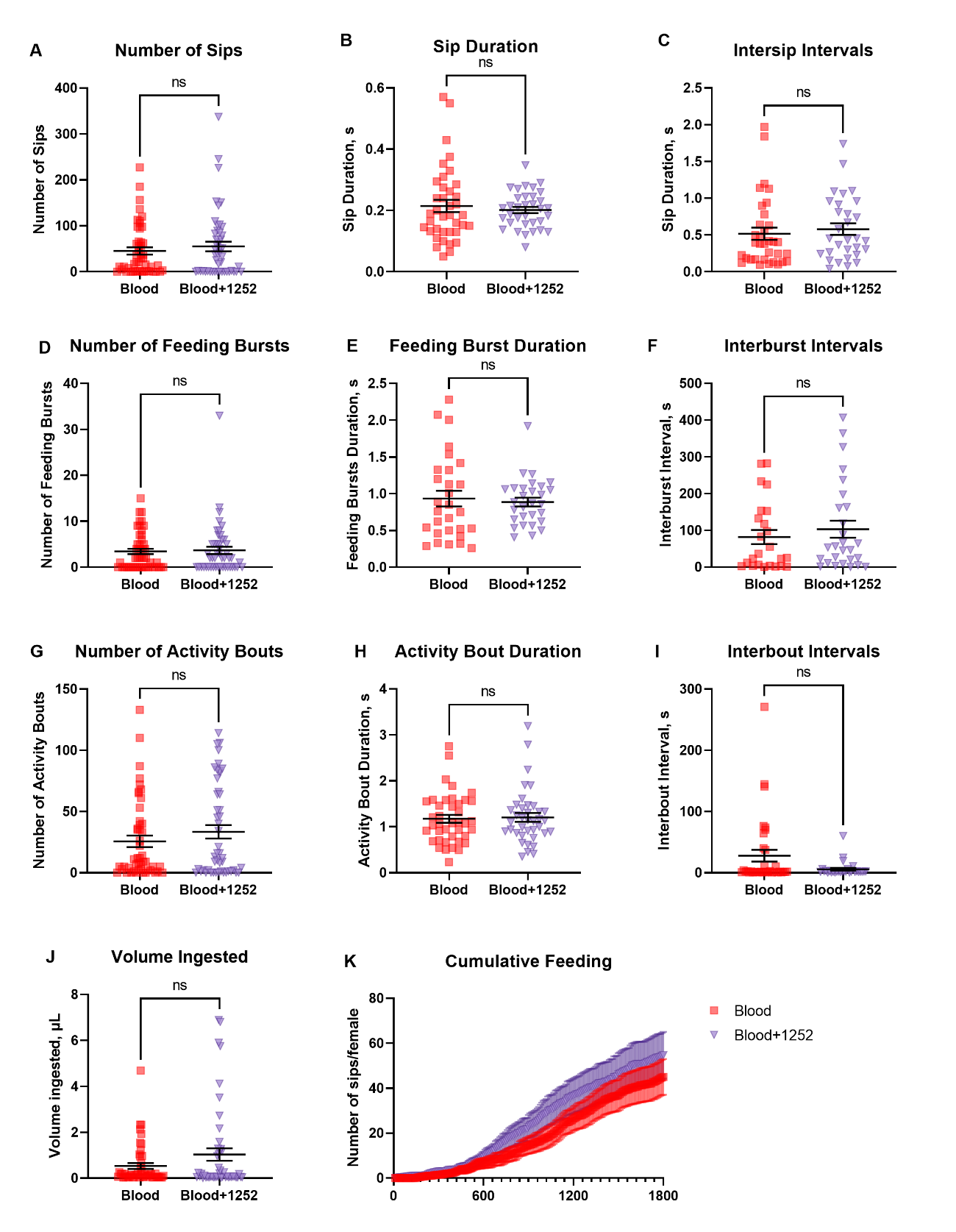


Panel 2: 10% Sucrose with molecule SACC-0121252. This partial antagonist of the *Ae. aegypti* kinin receptor did not alter any of the feeding variables analyzed by the flyPAD, except for a significant decrease in the feeding burst duration. The molecule did not affect the meal volume ingested.


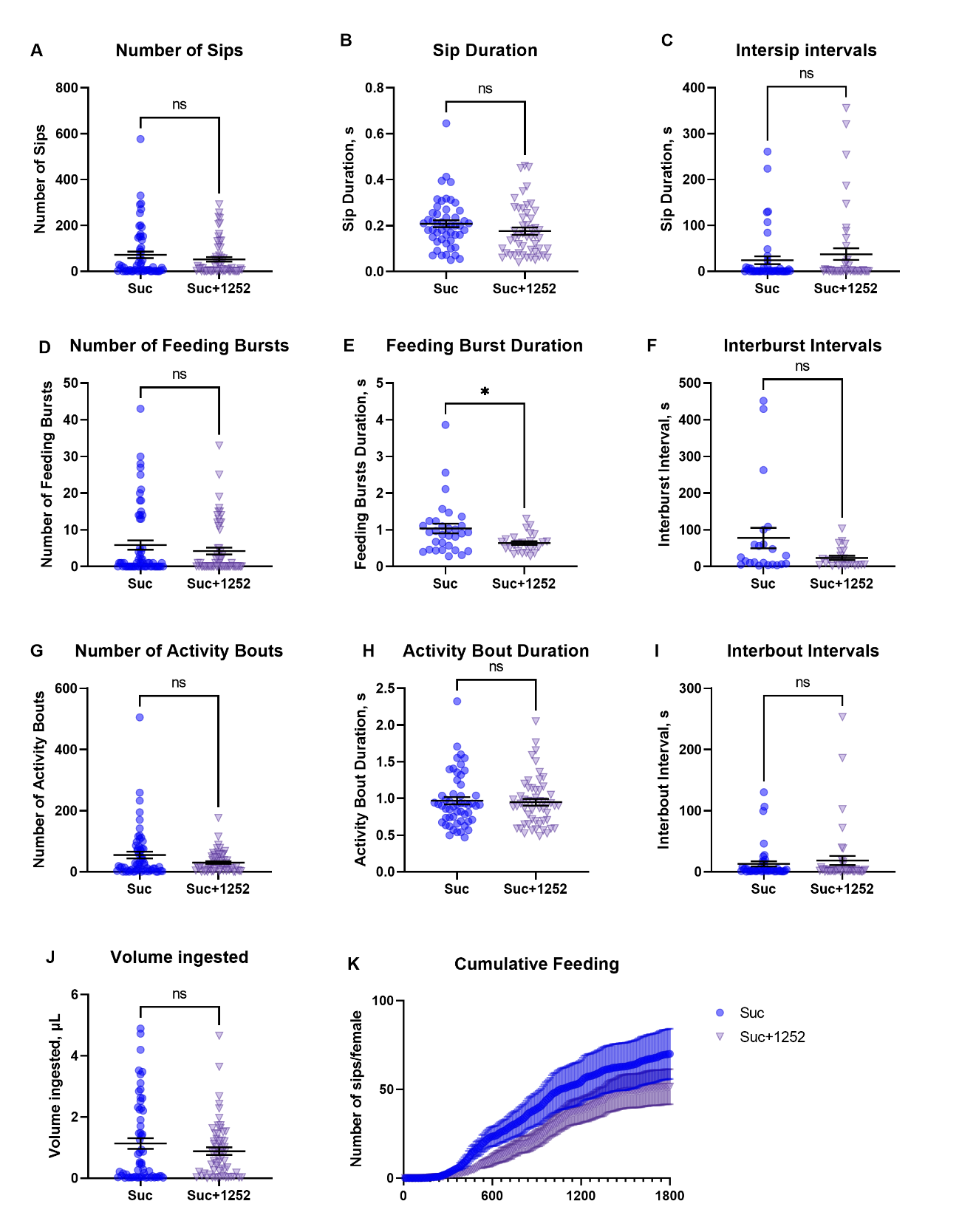


Panel 3. Blood with molecule SACC-0412060. This partial antagonist of the *Ae. aegypti* kinin receptor reduced the number of feeding bursts and the meal volume ingested. However, it did not alter any of the other feeding variables analyzed by the flyPAD.


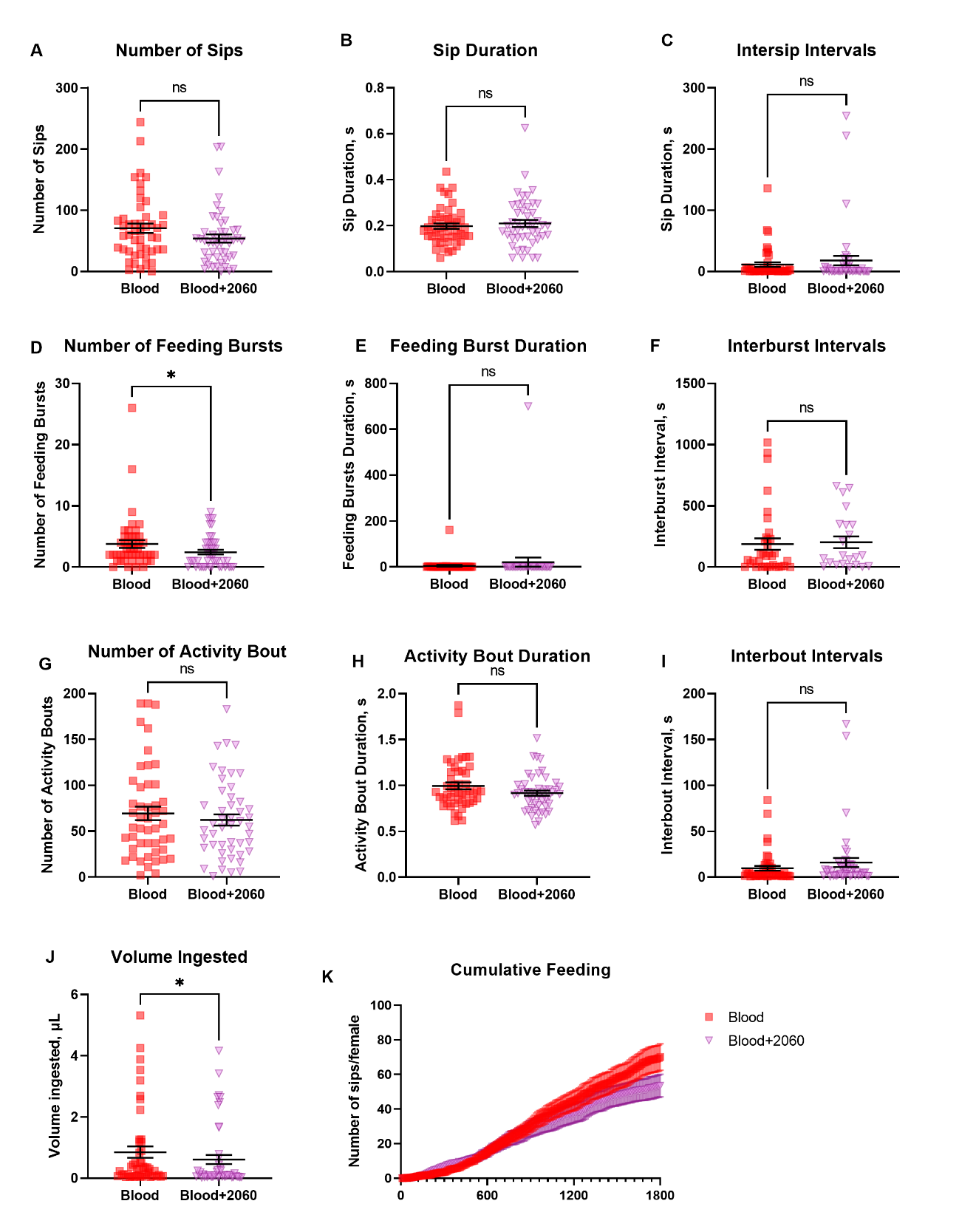


Panel 4. 10% Sucrose with molecule SACC-0412060. This partial antagonist of the *Ae. aegypti* kinin receptor reduced the sip and feeding burst durations and meal volume ingested. The molecule did not alter any of the other feeding variables analyzed by the flyPAD.


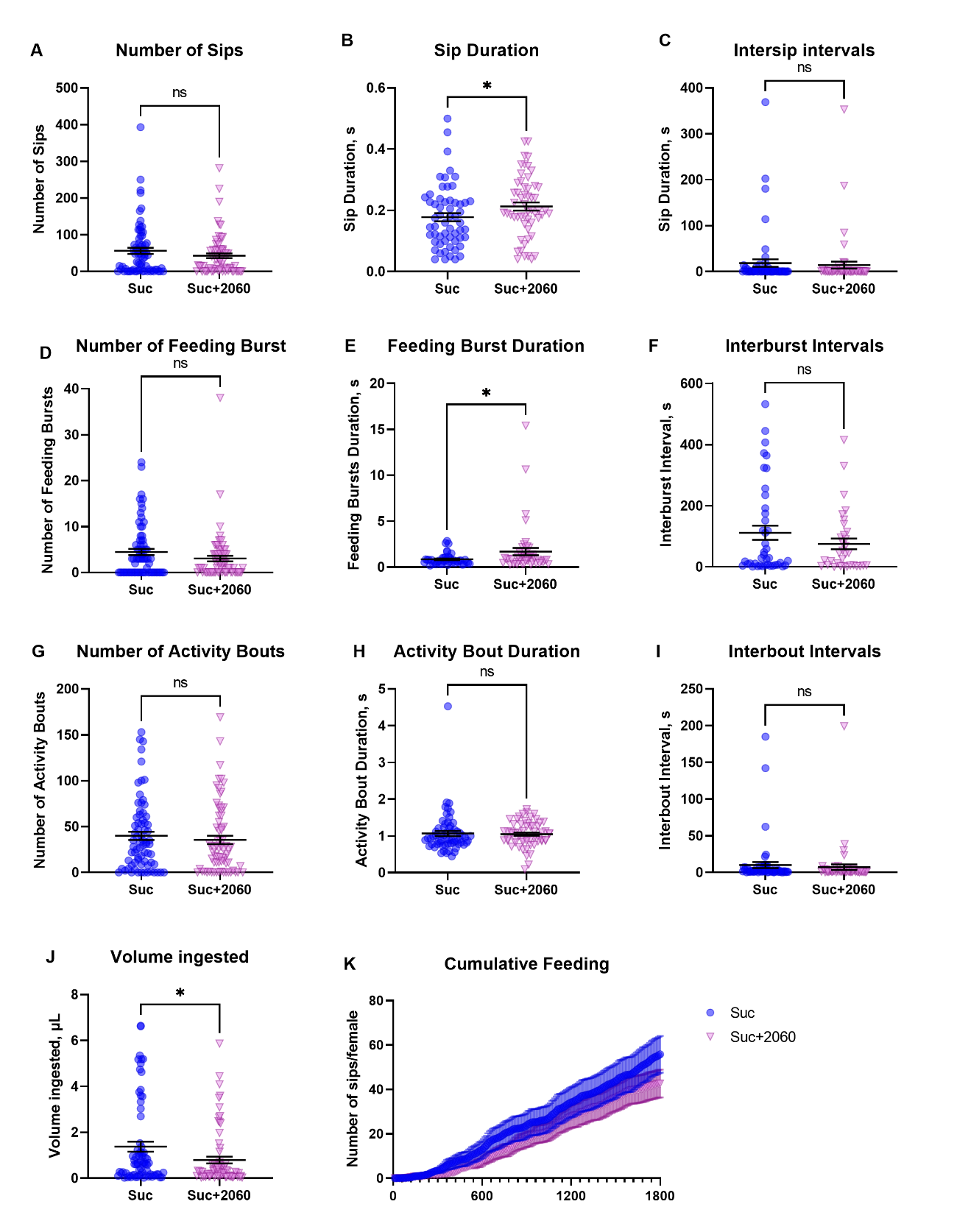


Panel 5. Blood with molecule SACC-0048555. This full antagonist of the *Ae. aegypti* kinin receptor did not alter any of the feeding variables analyzed by the flyPAD and did not affect the meal volume ingested.


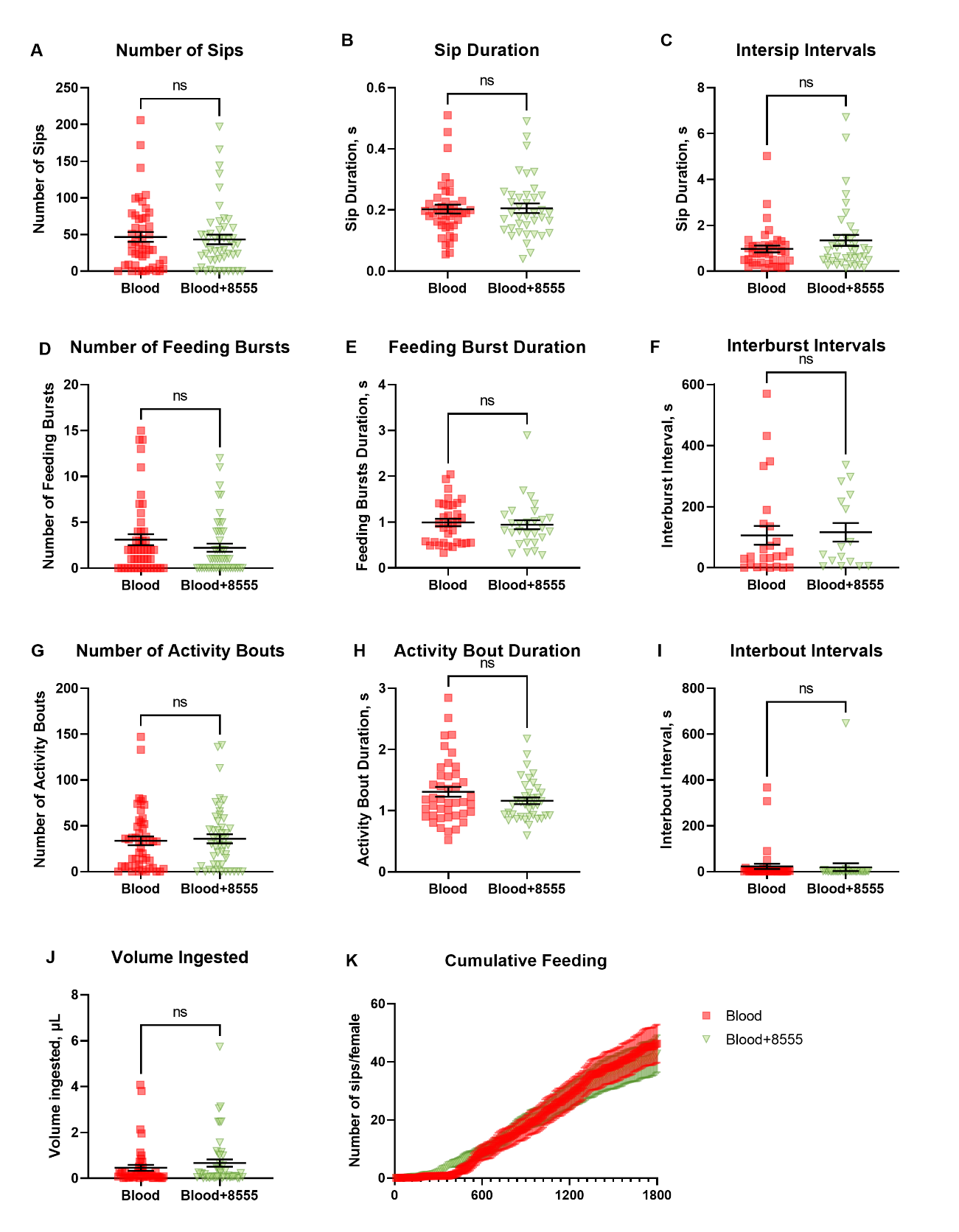


Panel 6. Blood with molecule SACC-0018618. This partial antagonist of the *Ae. aegypti* kinin receptor increased the intersip intervals and the feeding burst duration. The molecule did not alter any of the other feeding variables analyzed by the flyPAD and did not affect the meal volume ingested.


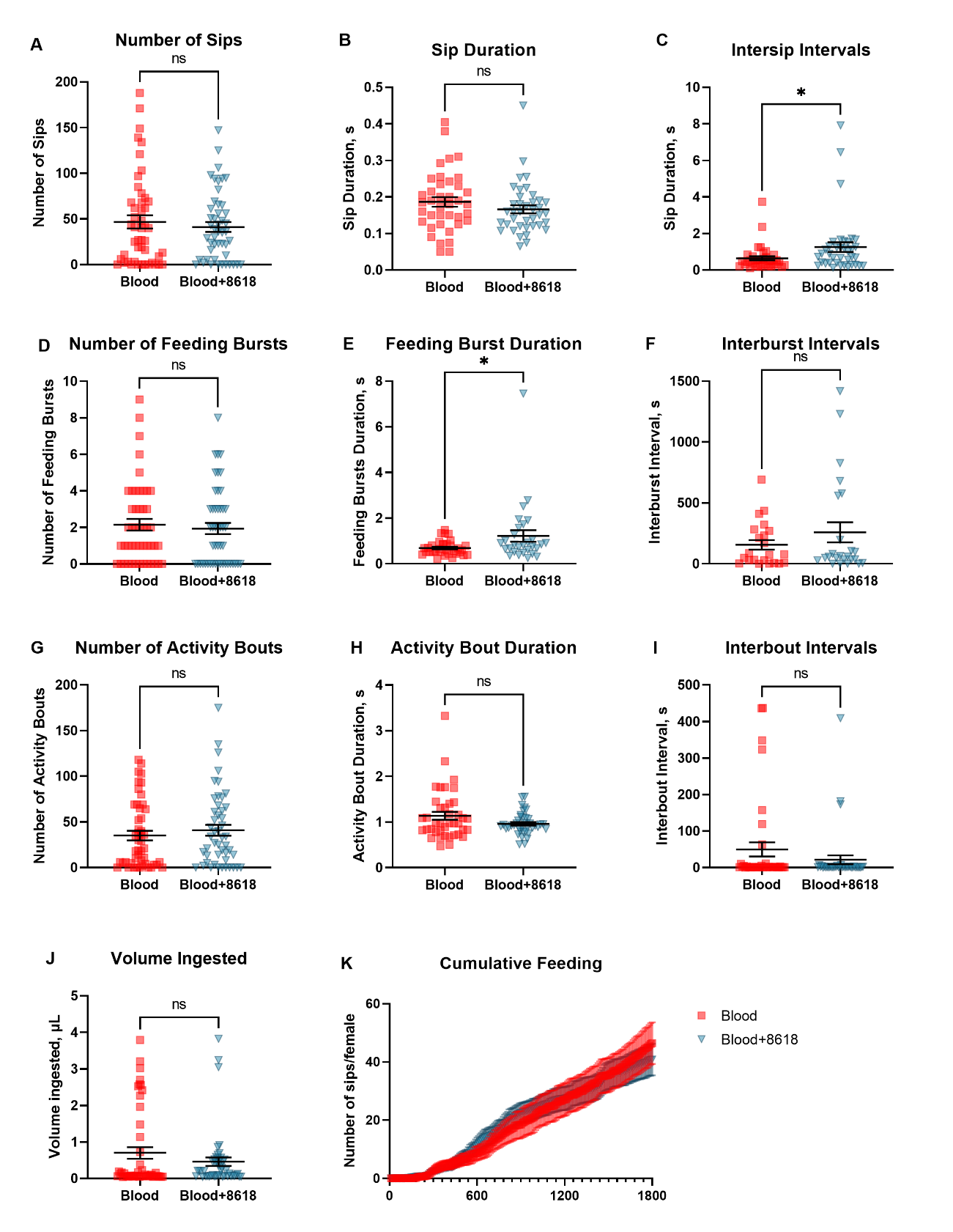


Panel 7. 10% Sucrose with molecule SACC-0018618. This partial antagonist of the *Ae. aegypti* kinin receptor increased the intersip intervals and the feeding burst duration. The molecule did not alter any of the other feeding variables analyzed by the flyPAD and did not affect the meal volume ingested.


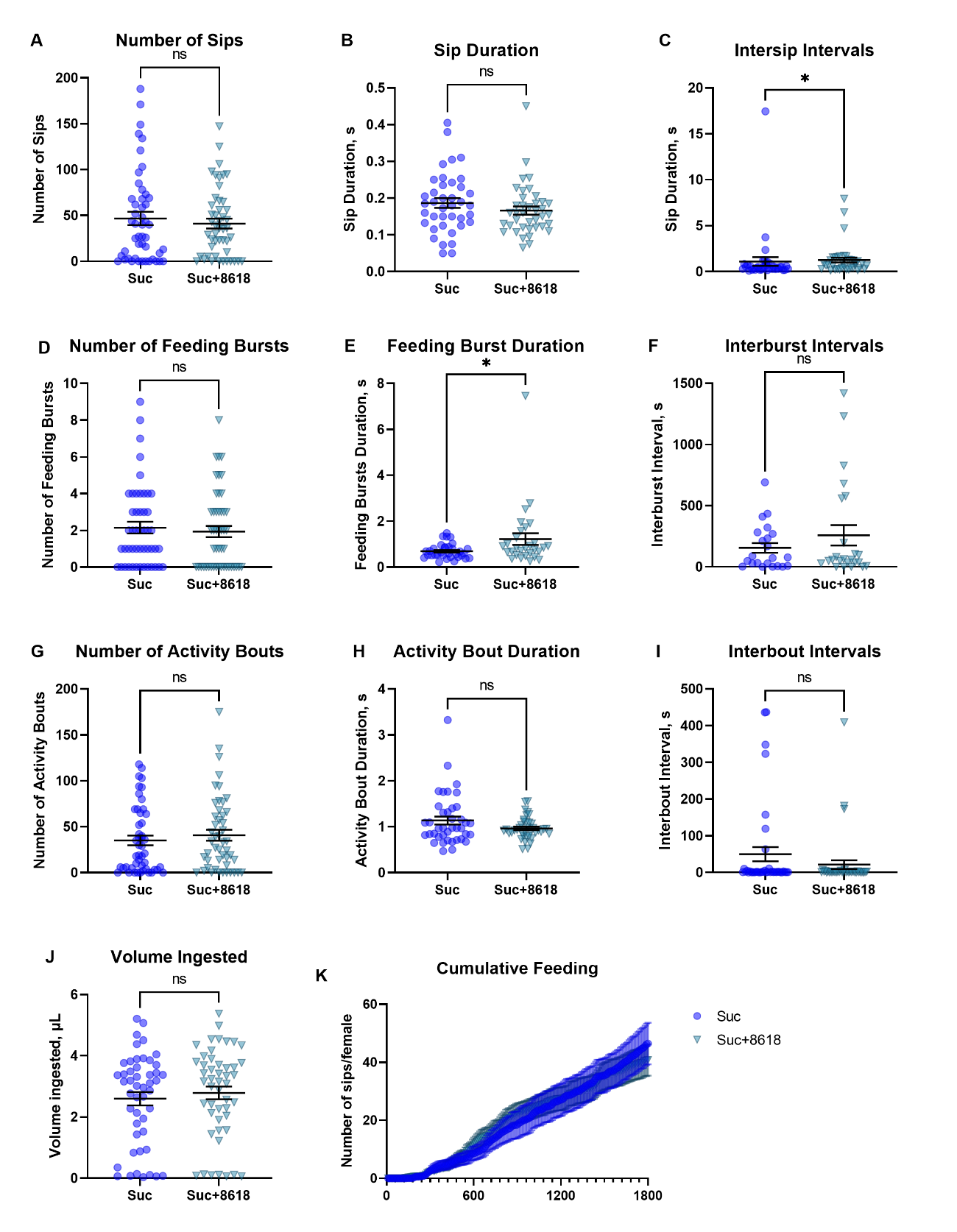


Panel 8. Blood with molecule SACC-0428768, belonging to the structural family of molecule SACC-0048555. This full antagonist of the *Ae. aegypti* kinin receptor did not alter any of the feeding variables analyzed by the flyPAD, except for a significant increase in the interbout intervals, and did not affect the meal volume ingested.


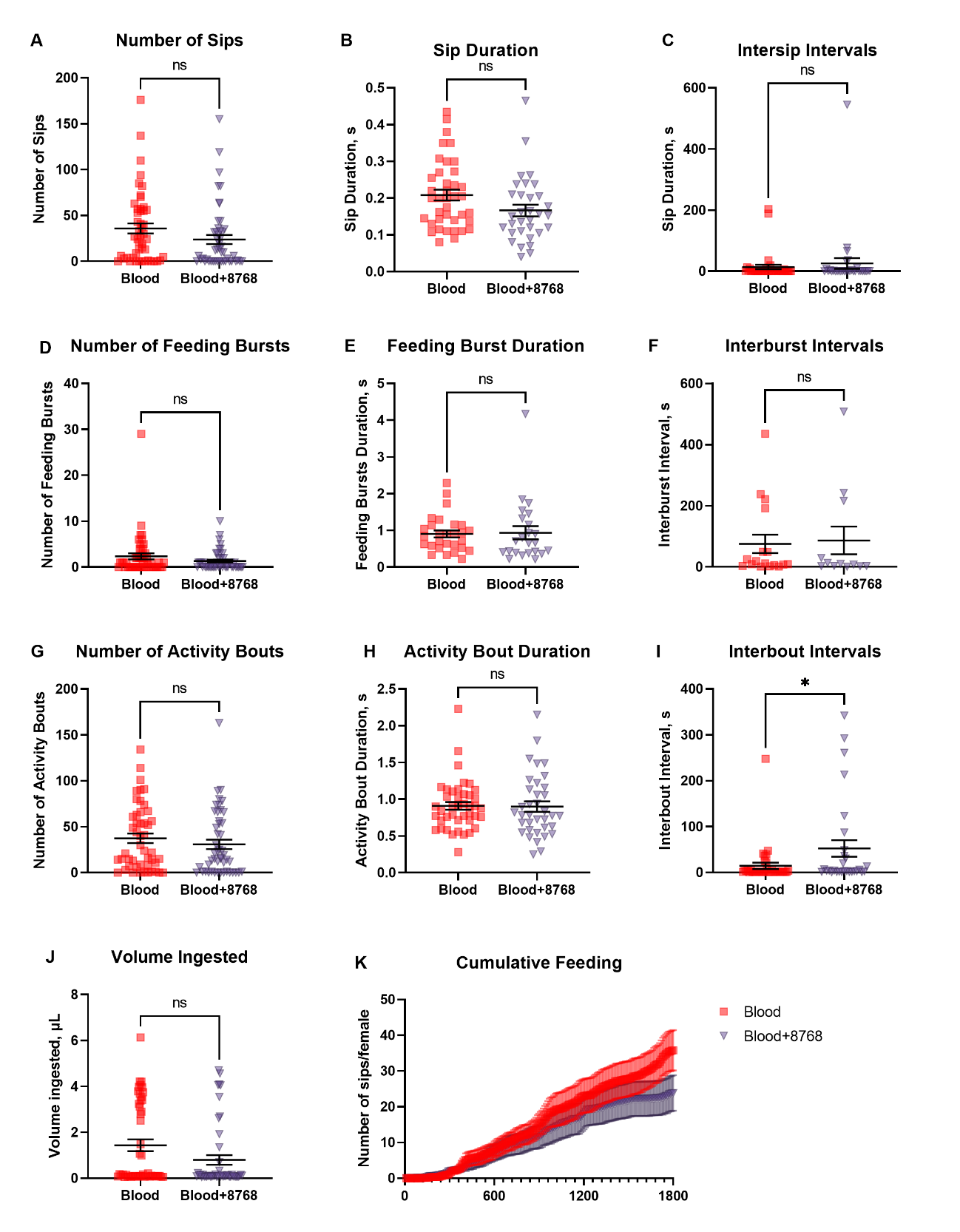


Panel 9. 10% Sucrose with molecule SACC-0428768, belonging to the structural family of molecule SACC-0048555. This full antagonist of the *Ae. aegypti* kinin receptor reduced the number of sips and of activity bouts, increased the sip and feeding burst durations, without affecting the meal volume ingested. The molecule did not affect any other variable analyzed by flyPAD.


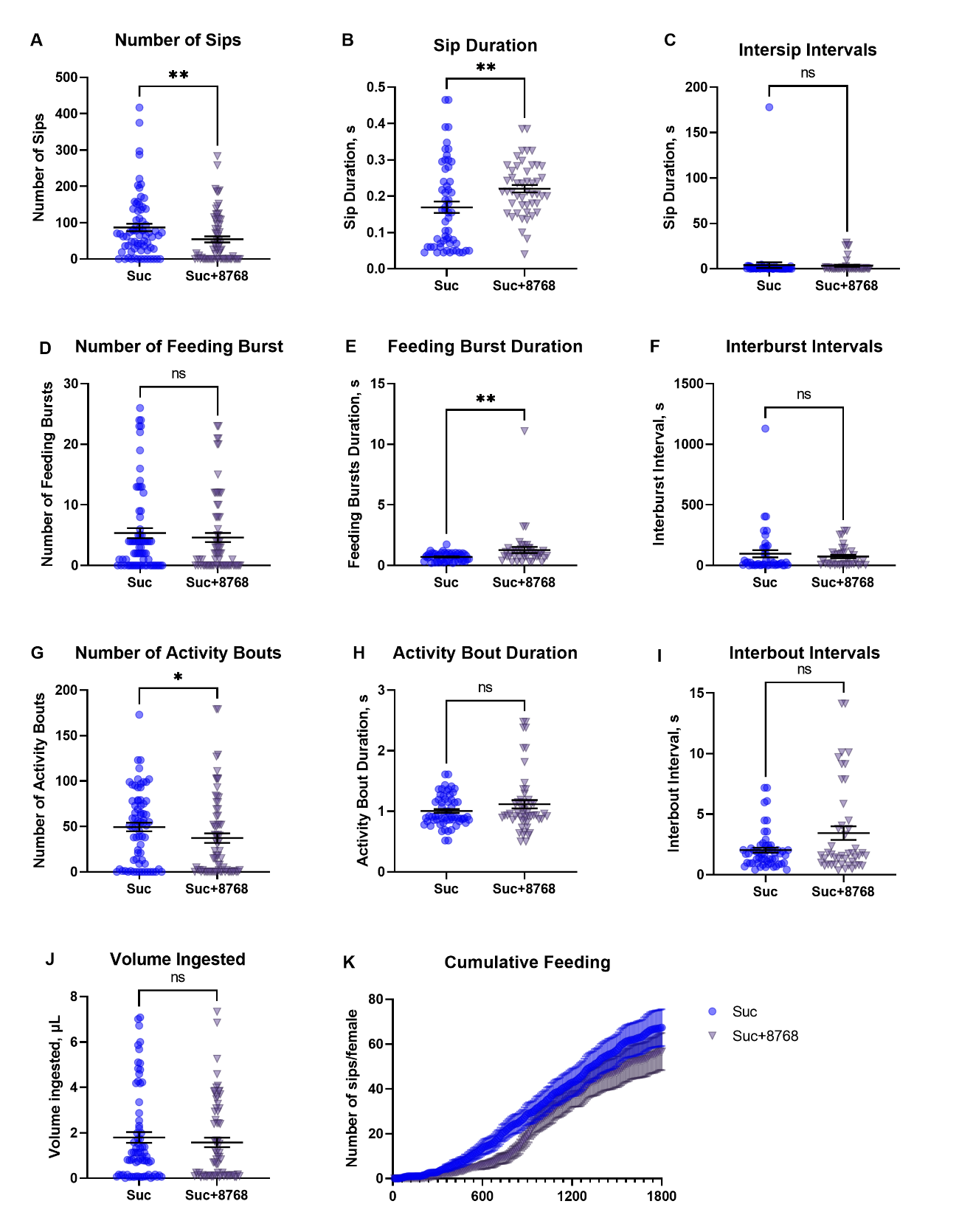


Panel 10. Blood with molecule SACC-0428771, belonging to the structural family of molecule SACC-0048555. This full antagonist of the *Ae. aegypti* kinin receptor did not alter any of the feeding variables analyzed by the flyPAD, except for a significant decrease in the number of activity bouts, and did not significantly affect the meal volume ingested.


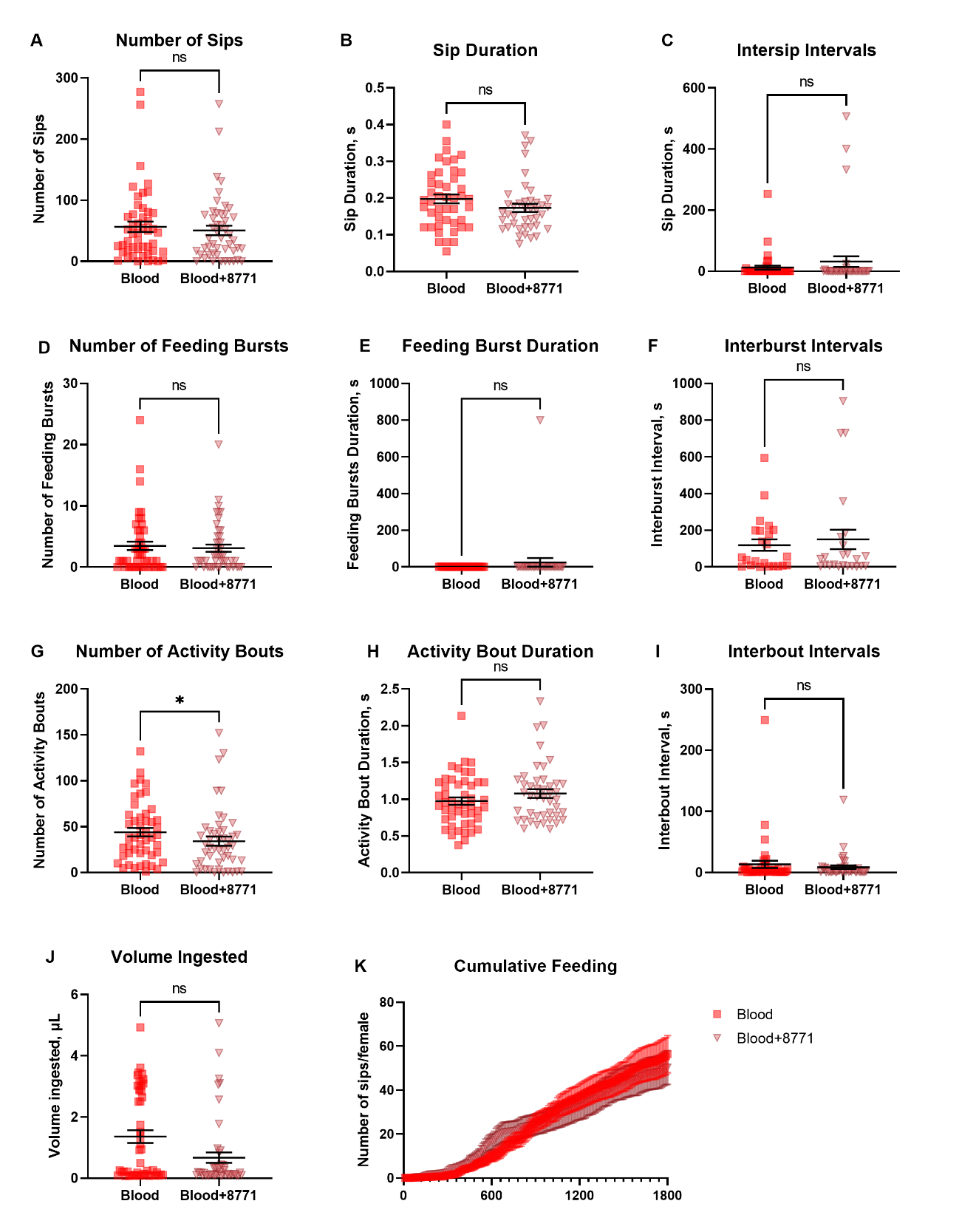


Panel 11. 10% Sucrose with molecule SACC-0428771, belonging to the structural family of molecule SACC-0048555. This full antagonist of the *Ae. aegypti* kinin receptor significantly decreased the number of activity bouts and the meal volume ingested. No other variable analyzed by flyPAD was affected.


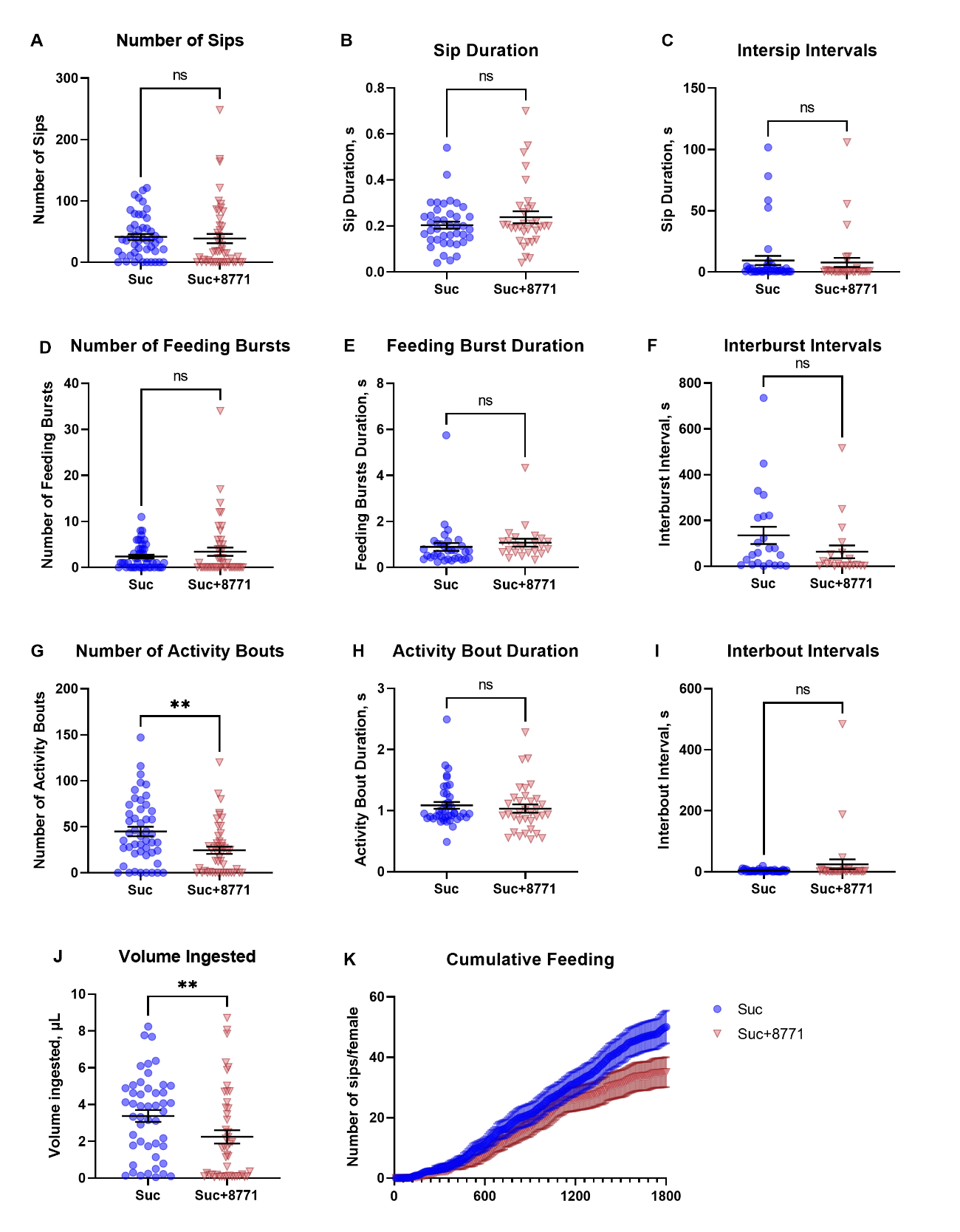


Panel 12. Blood with molecule SACC-0428773. This full antagonist of the *Ae. aegypti* kinin receptor did not alter any of the feeding variables analyzed by the flyPAD and did not affect the meal volume ingested.


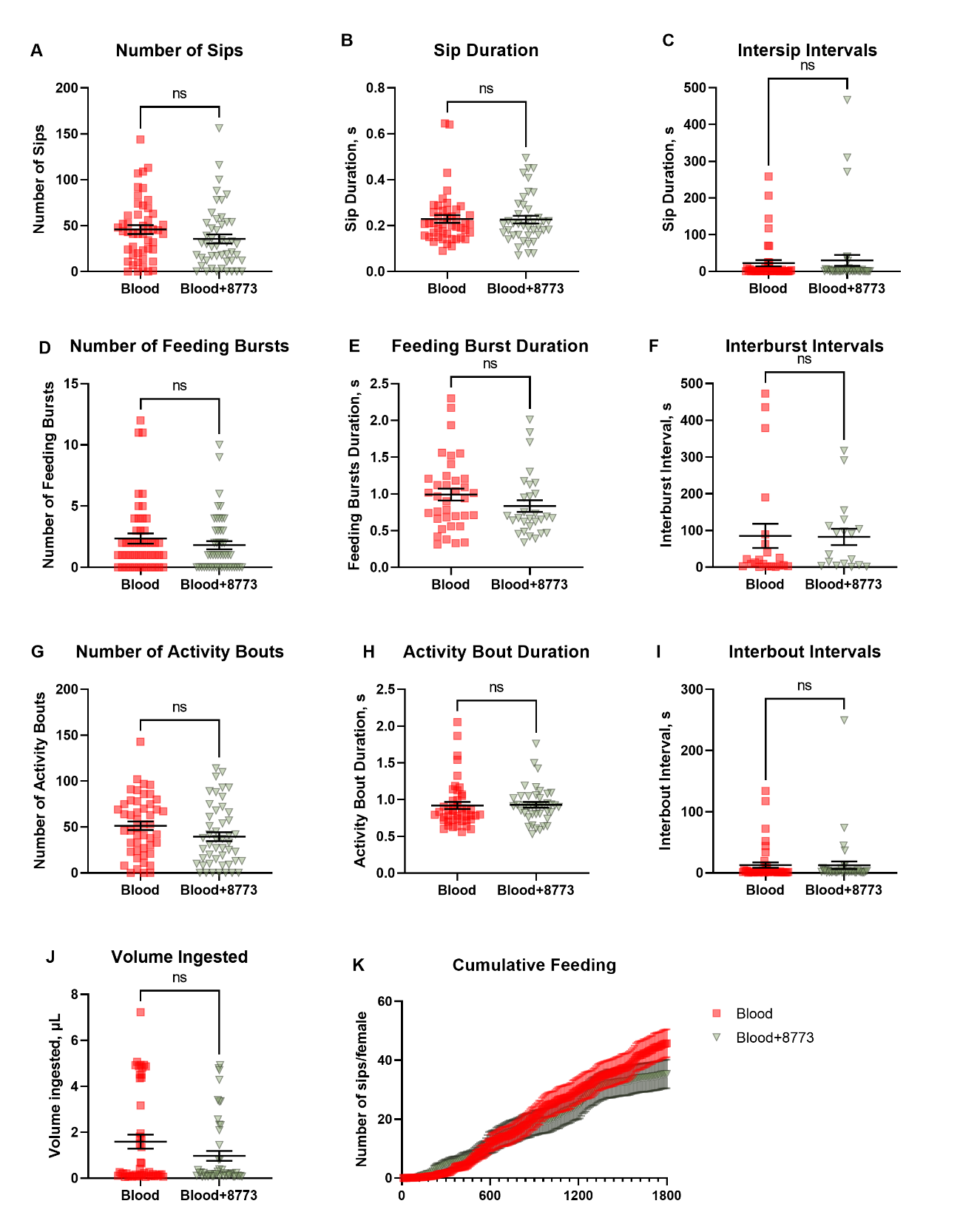


Panel 13. 10% Sucrose with molecule SACC-0428773. This full antagonist of the *Ae. aegypti* kinin receptor did not alter any of the feeding variables analyzed by the flyPAD, except for a significant increase in the sip duration, and did not affect the meal volume ingested.


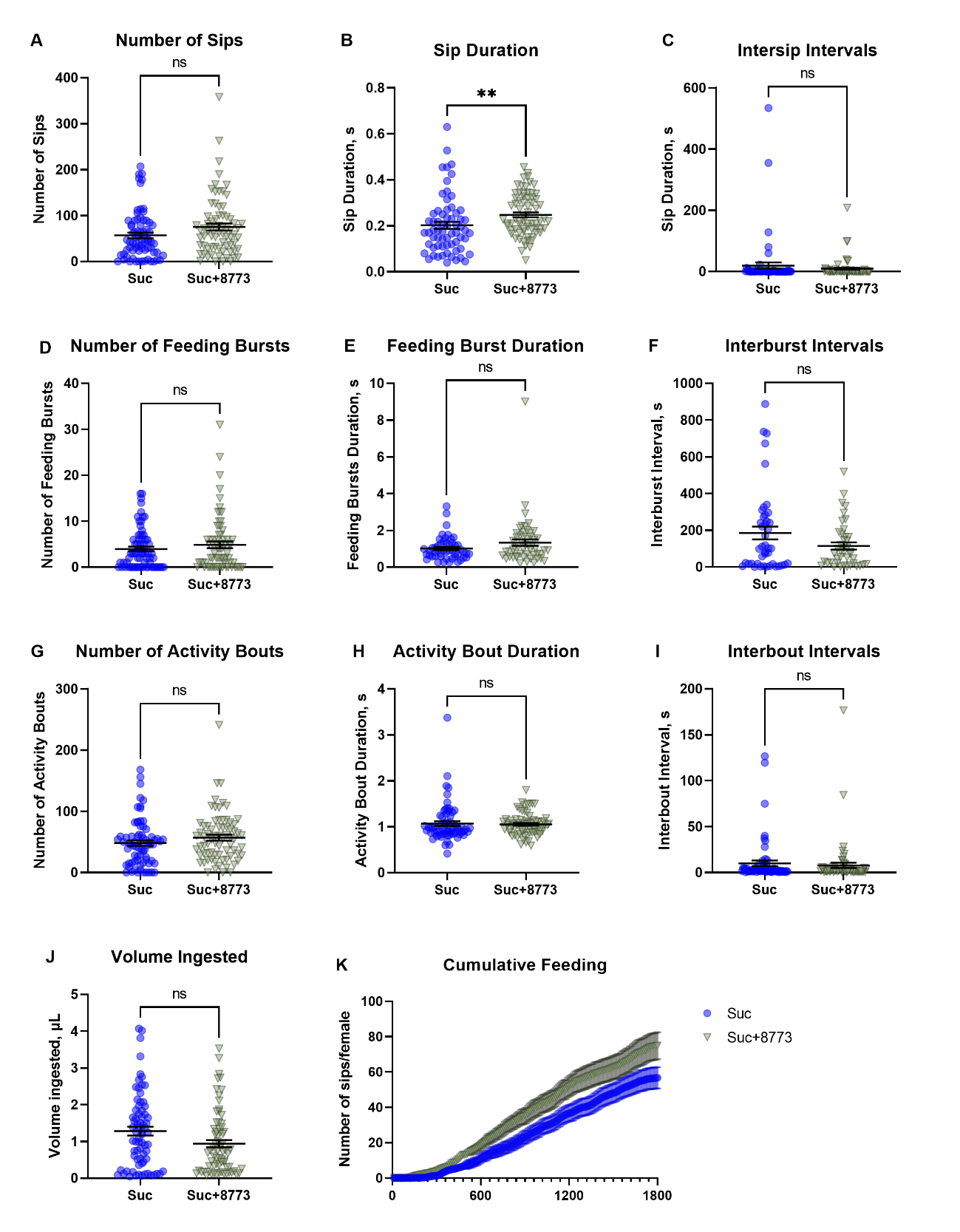


Panel 14. Blood with molecule SACC-0428774. This full antagonist of the *Ae. aegypti* kinin receptor did not alter any of the feeding variables analyzed by the flyPAD and did not affect the meal volume ingested.


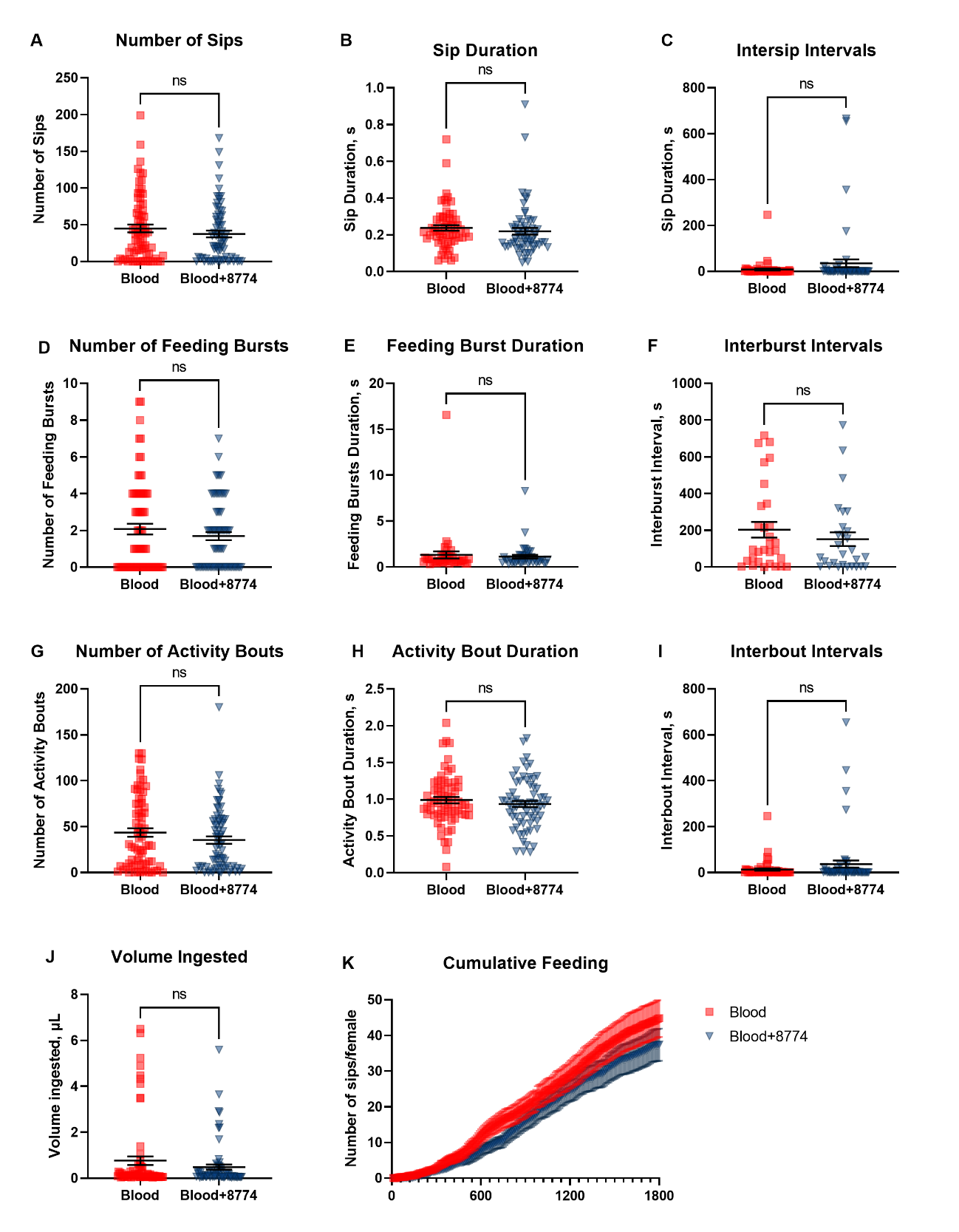


Panel 15. 10% Sucrose with molecule SACC-0428774. This full antagonist of the *Ae. aegypti* kinin receptor did not alter any of the feeding variables analyzed by the flyPAD, except for a significant increase in the intersip intervals and a decrease in the interburst intervals. Meal volume ingested was not affected.


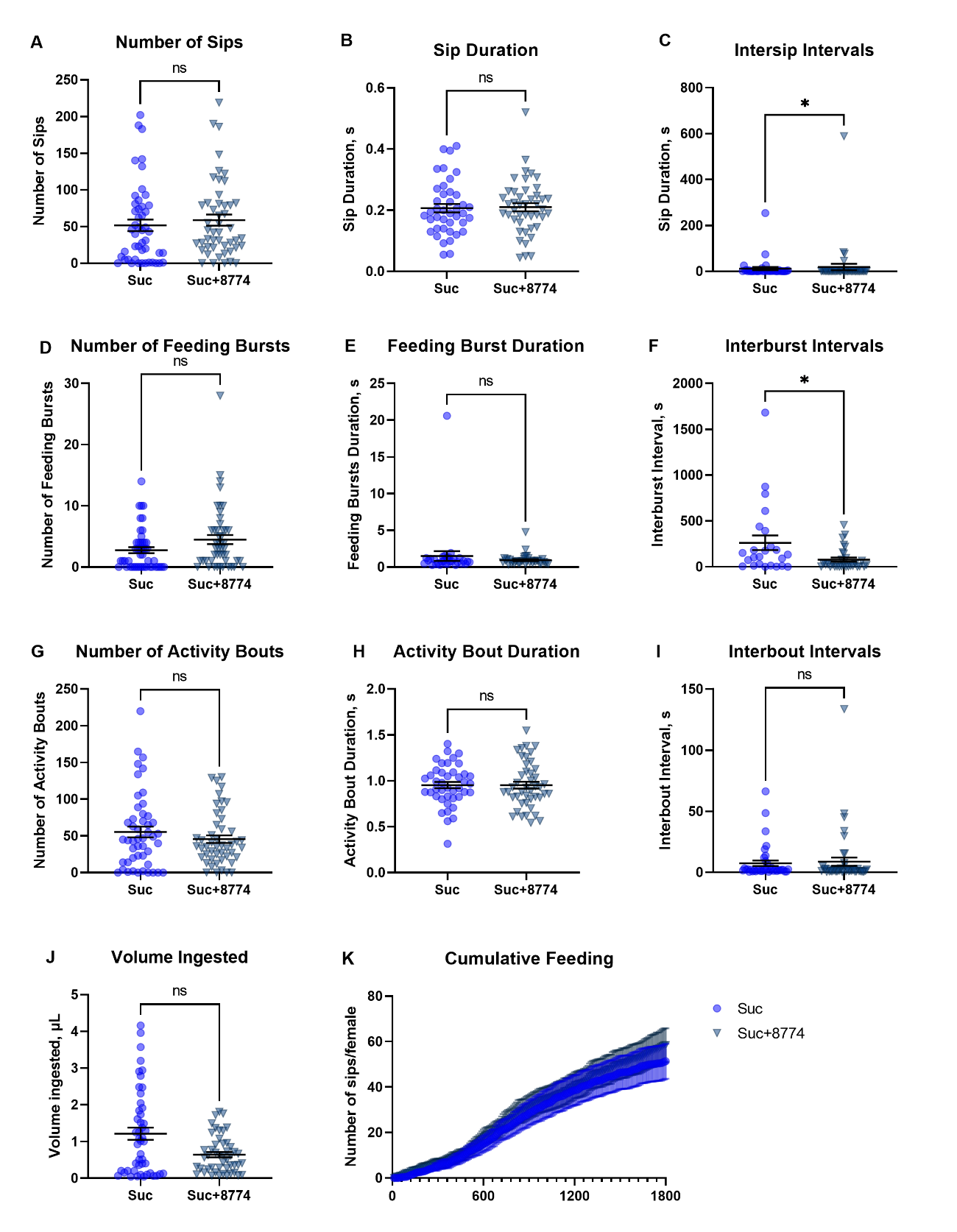


Panel 16. Blood with molecule SACC-0428775, belonging to the structural family of molecule SACC-0048555. This full antagonist of the *Ae. aegypti* kinin receptor did not alter any of the feeding variables analyzed by the flyPAD, except for a significant decrease in the number of activity bouts, and did not affect the meal volume ingested.


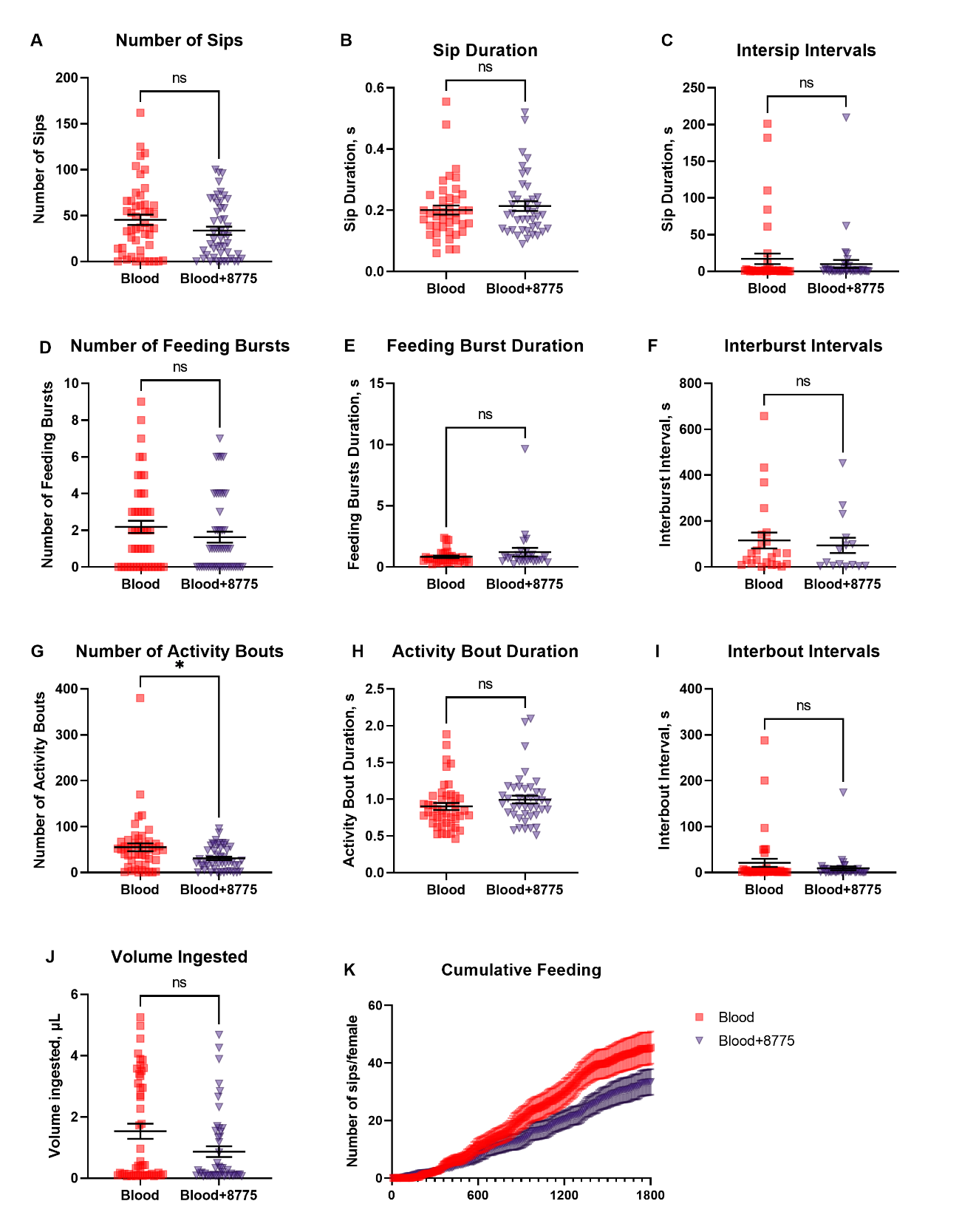


Panel 17. 10% Sucrose with molecule SACC-0428775, belonging to the structural family of molecule SACC-0048555. This full antagonist of the *Ae. aegypti* kinin receptor did not alter any of the feeding variables analyzed by the flyPAD, except for a significant increase in the sip and feeding burst durations. Meal volume ingested was not affected.


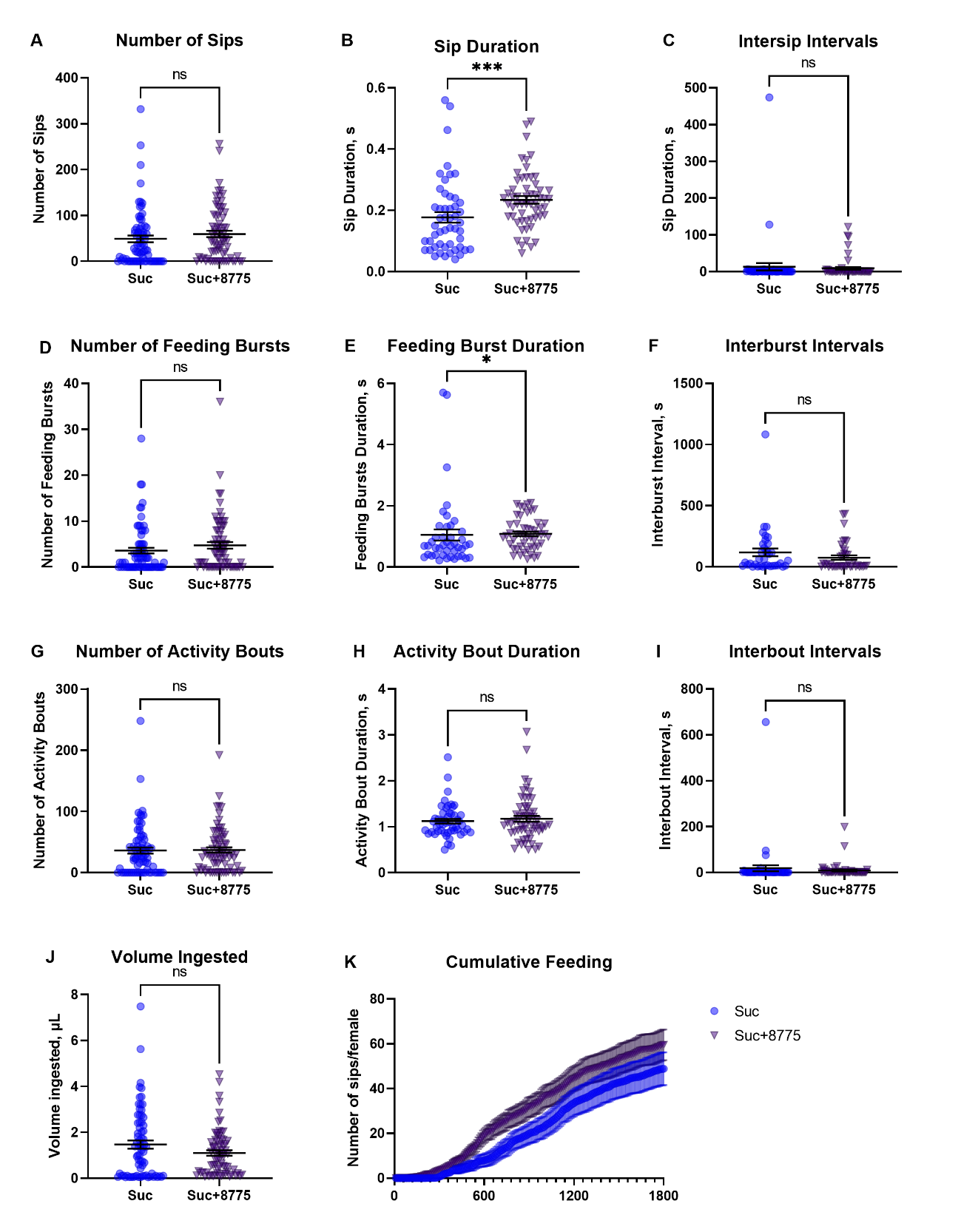


Panel 18. Blood with molecule SACC-0428796, belonging to the structural family of molecule SACC-0048555. This full antagonist of the *Ae. aegypti* kinin receptor did not alter any of the feeding variables analyzed by the flyPAD and did not affect the meal volume ingested.


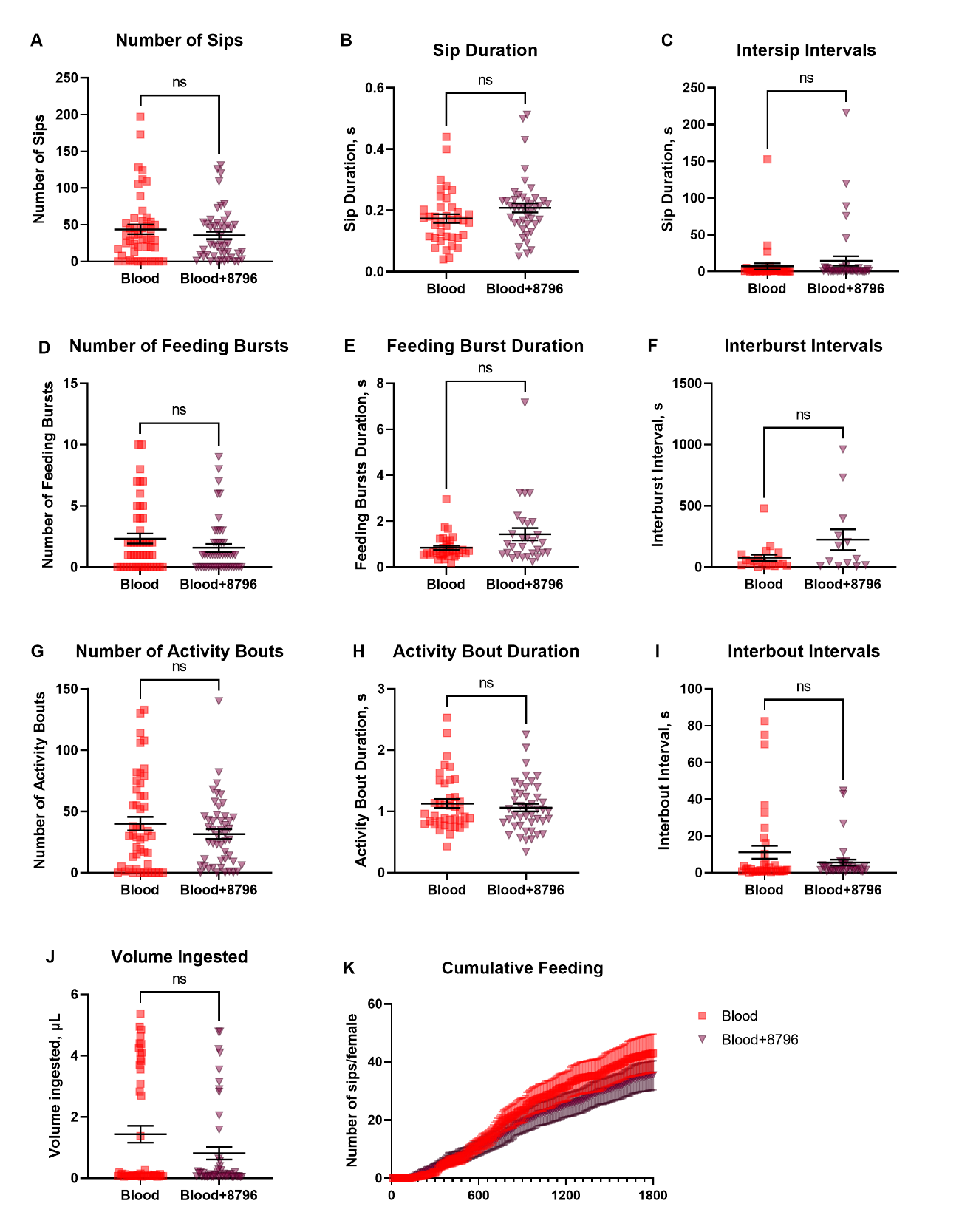


Panel 19. 10% Sucrose with molecule SACC-0428796, belonging to the structural family of molecule SACC-0048555. This full antagonist of the *Ae. aegypti* kinin receptor did not alter any of the feeding variables analyzed by the flyPAD, except for a significant decrease in the intersip intervals, and did not affect the meal volume ingested.


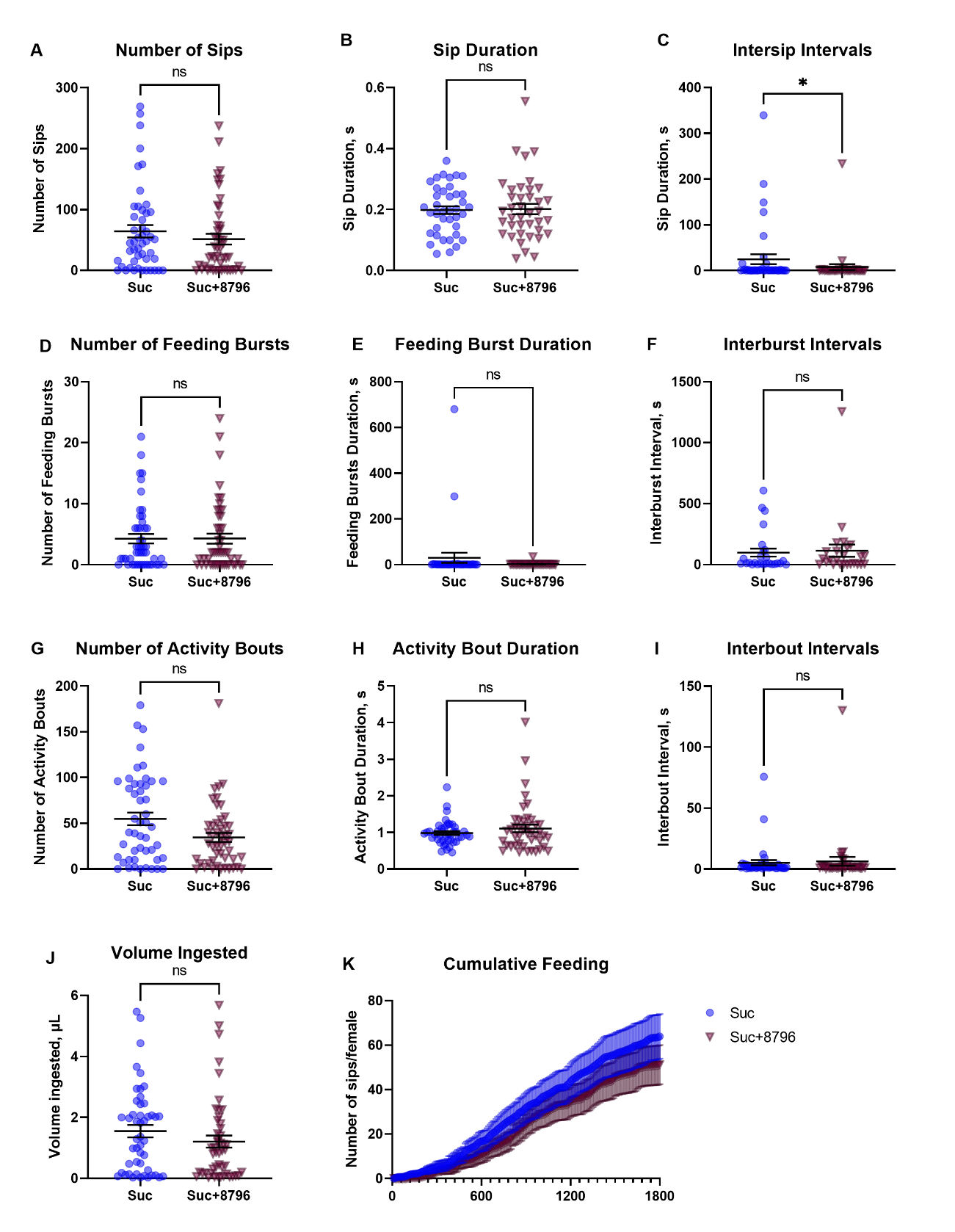


Panel 20. Sucrose with mosquitocidal molecule SACC-0039590. This molecule reduced sip durations and interburst interburst intervals, along with the meal volume ingested.
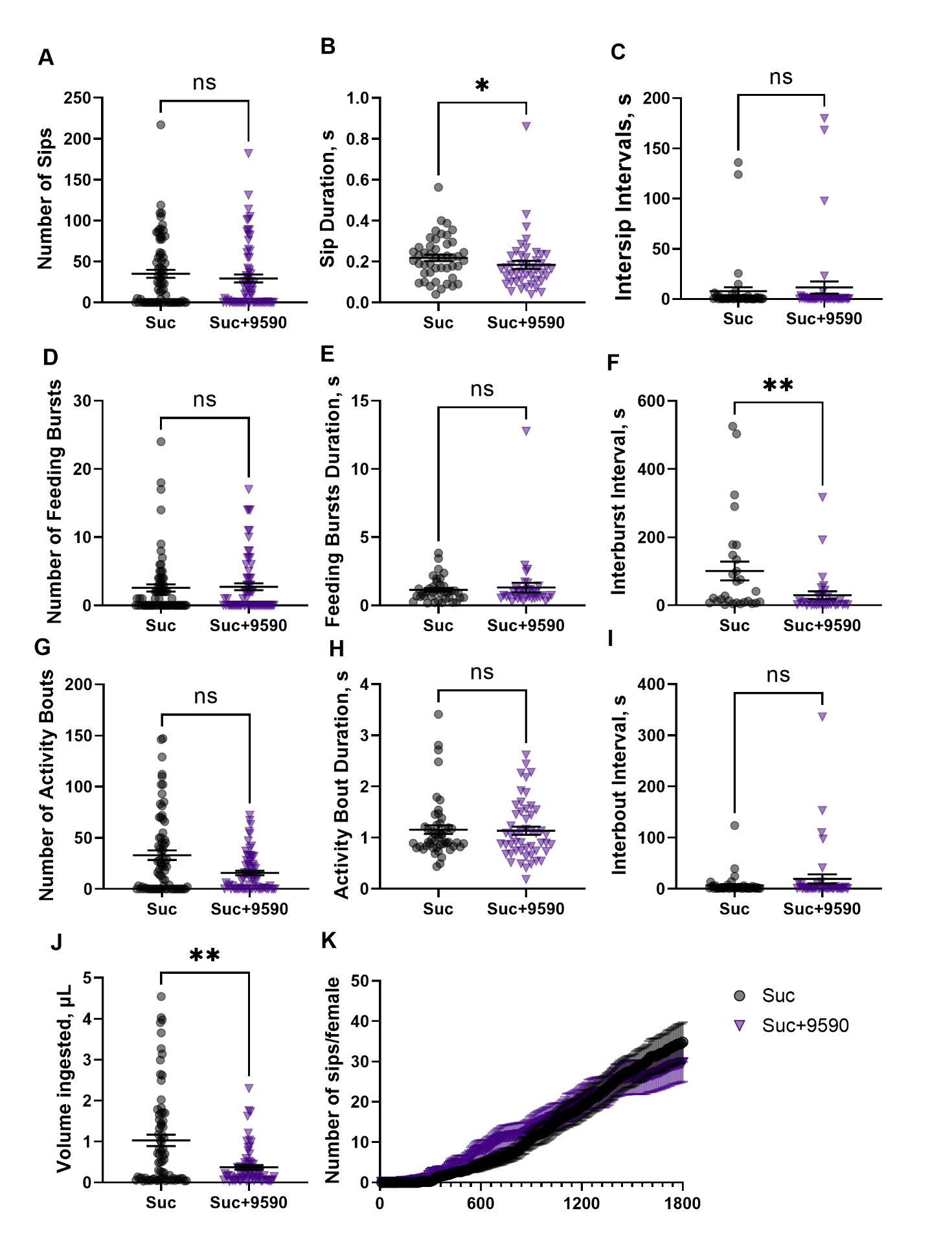

Supplement: Supplementary file 1 — Figure S1. FlyPAD assays with all antagonistic or mosquitocidal molecules in sucrose or blood. Comparison between feeding behaviors of female Aedes aegypti when offered control meals (blood or 10% sucrose) or the meal containing the test molecule at 1 mm (blood or suc + molecule number) using the flyPAD system in non‐choice assays. (A) Number of sips. (B) Duration of the sips (s). (C) Intersip intervals (s). (D) Number of feeding bursts, each characterized as three or more consecutive sips. (E) Duration of each feeding burst (s). (F) Duration of Interburst intervals (s). (G) Number of activity bouts, indicating how often the mosquito approaches the food. (H) Duration of the activity bouts (s). (I) Duration of interbout intervals (s). (J) Total volume ingested by each female. (K) Cumulative feeding, indicating the cumulative number of sips per female at every 10 s interval. Symbols represent outputs from individual mosquitoes, lines are mean ± standard error of the mean (SEM). Mann–Whitney test, asterisks denote a statistical significance, where one asterisk (*) indicates P < 0.05, and two asterisks (**) indicate P < 0.01, three asterisks (***) indicate P < 0.001, four asterisks (****) indicate P < 0.0001, and not significant (ns) indicates P > 0.05. [file PS-82-6842-s004.docx]
